# Identification of novel sRNAs involved in biofilm formation, motility, and fimbriae formation in *Escherichia coli*

**DOI:** 10.1038/srep15287

**Published:** 2015-10-15

**Authors:** Geunu Bak, Jungmin Lee, Shinae Suk, Daun Kim, Ji Young Lee, Kwang-sun Kim, Byong-Seok Choi, Younghoon Lee

**Affiliations:** 1 Department of Chemistry, KAIST, Daejeon 305-701, Korea; 2 Superbacteria Research Center, KRIBB, Daejeon 305-806, Korea

## Abstract

Bacterial small RNAs (sRNAs) are known regulators in many physiological processes. In *Escherichia coli,* a large number of sRNAs have been predicted, among which only about a hundred are experimentally validated. Despite considerable research, the majority of their functions remain uncovered. Therefore, collective analysis of the roles of sRNAs in specific cellular processes may provide an effective approach to identify their functions. Here, we constructed a collection of plasmids overexpressing 99 individual sRNAs, and analyzed their effects on biofilm formation and related phenotypes. Thirty-three sRNAs significantly affecting these cellular processes were identified. No consistent correlations were observed, except that all five sRNAs suppressing type I fimbriae inhibited biofilm formation. Interestingly, IS118, yet to be characterized, suppressed all the processes. Our data not only reveal potentially critical functions of individual sRNAs in biofilm formation and other phenotypes but also highlight the unexpected complexity of sRNA-mediated metabolic pathways leading to these processes.

Expression of about a hundred species of small RNAs (sRNAs) has been experimentally confirmed in *Escherichia coli*^1^,^2^,^3^,^4^,^5^,^6^,^7^,^8^. sRNAs interact with specific targets, such as protein, RNA or DNA to perform their respective functions, and have received significant attention as central regulators in a variety of cellular processes^9^,^10^,^11^,^12^,^13^,^14^,^15^. The majority of functionally characterized sRNAs base-pair with mRNA targets, thereby affecting mRNA stability and/or translational activity. Approximately one-third of the sRNAs identified to date strongly interact with Hfq protein, which enhances sRNA stability and facilitates hybridization to their target mRNAs^16^,^17^,^18^.

Bacteria can establish biofilm, a structure comprising multicellular aggregates embedded in extracellular matrix that adhere to each other and/or surfaces to protect against damaging environments. Biofilm formation occurs from both biotic and abiotic surfaces, thereby leading to severe pathogenic and industrial problems, such as increased antibiotic resistance, chronic infection, growth of bacteria in medical devices, and corrosion of industrial pipes^19^. Thus, the decision-making process of bacteria to develop biofilm or not should be precisely and dynamically regulated in response to environmental changes, owing to the high energy costs and irreversibility of the process^20^,^21^. Development of biofilm is one of the most complex physiological processes in bacteria. Several genes required for biofilm development have been investigated via microarrays^22^,^23^,^24^ and systematic knockout mutant studies^25^. Generally, flagella-based motility, cell surface appendages, such as type I or curli fimbriae, lipopolysaccharide (LPS), other genes related to the cell membrane, and exopolysaccharide (EPS) are considered important factors for initial attachment of cells and maturation of complex biofilm structure^26^.

sRNAs usually respond to specific environmental stress conditions and regulate a number of genes participating in stress adaptation^15^,^27^. Moreover, many sRNAs modulate outer membrane or surface proteins in *E. coli* and/or *Salmonella enterica*^28^,^29^. Accordingly, it is speculated that sRNAs are involved in regulation networks linking environmental cues and metabolic changes during development of biofilm. Since biofilm formation is a phenotype that could be generated from comprehensive response to various cellular stress factors, the regulatory mechanisms involved remain poorly understood. Therefore, collective analysis of sRNAs affecting biofilm formation and related phenotypes may provide insights into the cellular mechanisms underlying this process. Previously, the group of Gottesman examined the effects of a collection of plasmids overexpressing 26 Hfq-dependent sRNAs on *rpoS* and *flhDC* expression and swimming motility^30^,^31^. Although the collection only contained part of the sRNAs experimentally validated in *E. coli*, this approach led to the identification of both previously identified and novel sRNAs. For instance, in addition to two previously defined sRNAs (DsrA and RprA), ArcZ was identified as a sRNA involved in *rpoS* activation, McaS as an sRNA activator, and ArcZ, OmrA, OmrB, and OxyS as negative regulators of *flhDC* expression and swimming motility.

sRNAs controlling expression of *flhDC, rpoS* or *csgD* (encoding the key biofilm regulator), such as ArcZ, DsrA, RprA, McaS, OmrA/OmrB, and GcvB, have been shown to affect biofilm formation^32^. While sRNAs affecting motility through changes in flagella expression appear to affect biofilm formation, this finding cannot be generalized, since only a limited number of sRNAs have been examined to date. In the current study, we expressed 99 experimentally verified sRNAs, with the aim of evaluating their effects on biofilm development processes and establishing the relationship between motility and biofilm formation. In terms of motility, we examined the effects of sRNAs, not only on swimming but also swarming motility. Other biofilm related-phenotypes, such as type I and curli fimbriae formation, were additionally assessed.

Overall, we identified 33 sRNAs that significantly affect biofilm formation and related phenotypes of swimming and swarming motilities, type I fimbriae or curli fimbriae formation. However, no consistent correlations among these were evident, except that all five sRNAs suppressing type I fimbriae formation also inhibited biofilm formation. Even two homologous sRNAs, OmrA and OmrB, which were previously reported as repressors of curli formation^33^, induced different swimming and swarming motility phenotypes. Interestingly, IS118, which has not been characterized as yet, suppressed all the processes examined. We also identified new sRNAs, such as CsrB, DicF, GadY, IS118, Och5, SdsR and SgrS, which directly or indirectly target biofilm-related genes *csgD, flhD* and/or *pgaA*. Our collective findings disclose potentially critical individual sRNAs involved in biofilm formation or related phenotypes and highlight the unexpected complexity of sRNA-mediated metabolic pathways leading to these processes.

## Results

### Construction of plasmids expressing sRNAs

**Figure 1:**
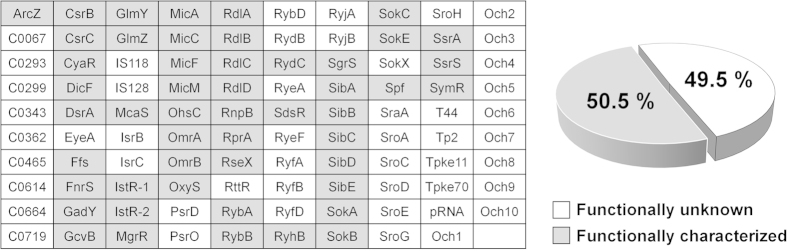
*E. coli* small RNAs used in this study. sRNA-expressing plasmids were constructed for 99 experimentally validated sRNAs. sRNAs whose functions were previously characterized are shown in gray. Full references for each sRNA were provided in Supplementary Table 1.

Although several hundred sRNAs have been predicted in *E. coli*, only ~100 have been experimentally validated using northern blot, microarray, and RNA-seq analyses to date^1^,^2^,^3^,^4^,^5^,^6^,^7^,^8^. Several of the experimentally verified sRNAs have not been functionally characterized as yet (Fig. 1, Supplementary Table 1). Here, we overexpressed a collection of sRNAs, with a view to characterizing their physiological functions in biofilm formation, motility and fimbriae formation. To this end, we constructed plasmids overexpressing 99 experimentally validated sRNAs via IPTG induction. The sRNA sequences were cloned into the RNA expression vectors pHM-tac, pHMB1 or pHMB2 (Supplementary Fig. 1). For sRNAs with known 5′ ends, we designed expression plasmids to facilitate transcription from this start site. However, if the 5′ end was not adenine, an extra adenine residue was added to the 5′ end of sRNAs to facilitate efficient transcription. In cases where 5′ ends were only predicted, ~20 nt upstream chromosomal sequences were added. For the 3′ end, >25 nt downstream chromosomal sequences were added to the predicted 3′ end or the identified termination site. If the cloned fragment did not contain a termination signal, transcriptional termination could occur at the *rnpB* terminator adjacent to the cloning site. The full sequences of cloned fragments for sRNA expression are presented in Supplementary Table 2.

**Figure 2:**
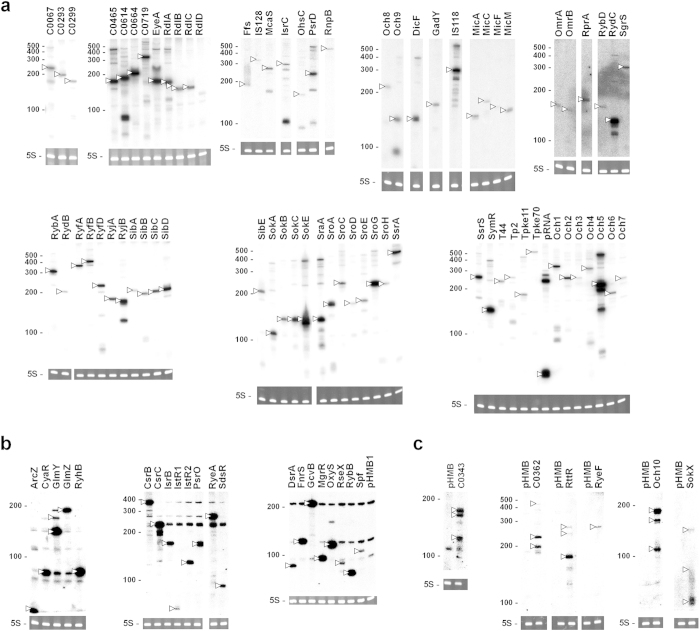
Northern blot analysis of overexpressed sRNAs. MG1655 cells containing each sRNA-expressing plasmid were grown to OD_600_ ~ 0.6, and induced with 1.0 mM IPTG for 20 min. Total RNAs (10 μg) were subjected to northern blot. Membranes were probed with rnpBXb1 (**a**), specific oligonucleotide mixes (**b**), or each specific oligonucleotide (**c**). RNA sizes were estimated using Century marker (Ambion), indicated on the left. 5S rRNA stained with ethidium bromide was also used as a loading control. Overexpressed sRNAs are marked by arrowheads.

To validate expression from RNA expression plasmids, total RNAs purified from 1 mM IPTG-induced cells transformed with individual plasmids were analyzed via northern blot. Since expressed RNAs contain the *rnpB* terminator sequence if a transcription termination signal is not included in the cloned fragment or does not lead to complete termination, we employed an oligonucleotide probe, rnpBXbI, complementary to the *rnpB* terminator. The rnpBXbI probe was used to successfully detect expression of 72 sRNAs (Fig. 2a). Owing to the presence of the *rnpB* terminator sequence, the observed lengths of expressed sRNAs were ~50 nt longer than the expected sizes. We additionally observed minor bands, which would be transcriptionally terminated further downstream, and their processed products. Expression of other sRNAs was analyzed with specific probes for each sRNA. To reduce the number of individual steps for northern analysis with specific probes, we applied a mixture of 5 to 8 different probes to one membrane. Using this protocol, expression of 21 sRNA species was verified (Fig. 2b). The expression of the remaining 6 species was validated with the corresponding probes (Fig. 2c). Ultimately, IPTG-induced expression of all 99 sRNAs was confirmed.

Interestingly, northern blot data using a mixture of probes revealed possible regulatory networks between some sRNAs. For instance, overexpression of RyeA and SdsR led to mutual reciprocal repression, which was also observed with CsrB and CsrC. In addition, increased expression of GlmZ was observed upon GlmY sRNA overexpression, consistent with a previous report^34^. All RNAs, except C0362 and RyeF, were detected with the expected sizes. C0362 sRNA, expected to yield a ~386 nt product, was detected as ~200 and ~230 nt bands (Fig. 2c), while RyeF, predicted as 388 nt, was detected as a ~280 nt species (Fig. 2c). The reasons underlying these discrepancies remain to be elucidated.

### Small RNAs involved in biofilm formation

**Figure 3:**
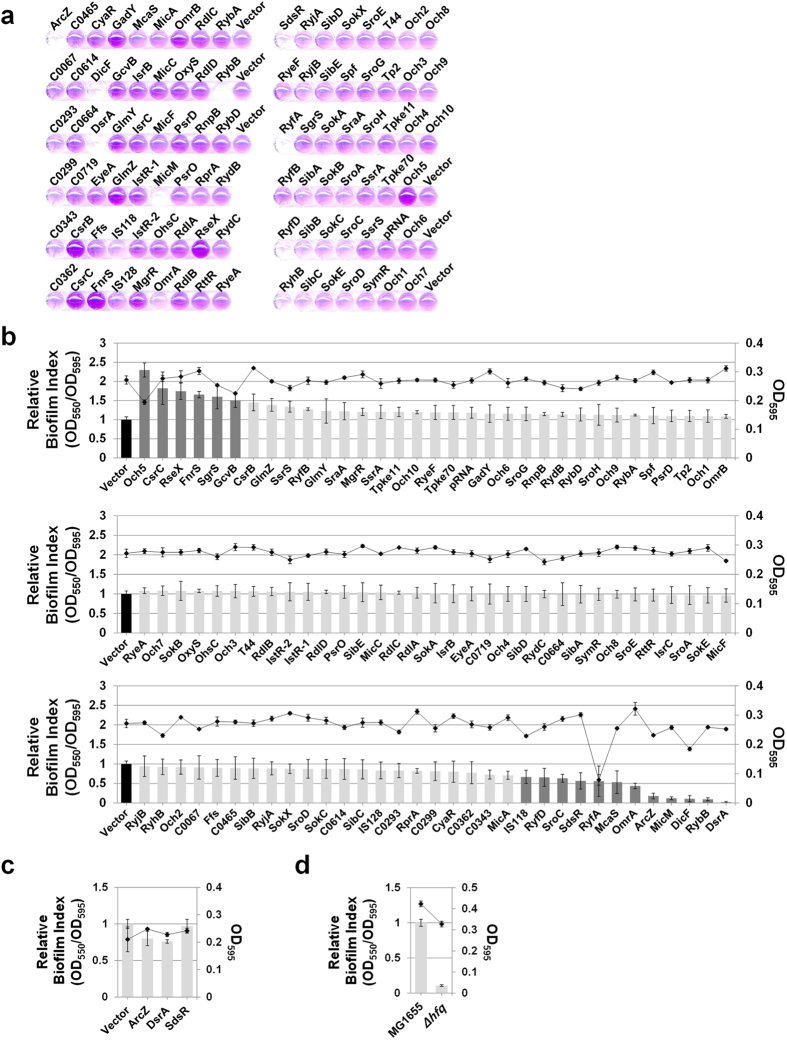
Effects of small RNA overexpression on biofilm formation in *E. coli*. (**a**) To characterize the biofilm forming ability of *E. coli* overexpressing sRNAs, a collection of strains containing each sRNA-expressing plasmid were grown in LB containing 1 mM IPTG and 100 μg/mL ampicillin at 30 °C for 12 h, and the amount of biofilm attached to 96-well round bottom polystyrene microtiter plates was measured via crystal violet staining. (**b**) The level of biofilm formation (OD_550_) was expressed relative to cell growth (OD_595_), and termed ‘biofilm index’. The biofilm index value was normalized to control cells containing plasmid vector (pHMB1) and designated ‘relative biofilm index’. (**c**) Effects of ArcZ, DsrA, and SdsR sRNAs on biofilm formation in the MG1655*Δhfq* background. (**d**) Comparison of relative biofilm formation by MG1655 and MG1655*Δhfq* strains. Relative biofilm indexes are shown on a bar graph (axis on the left) and the growth of each strain as a line graph (axis on the right).

Firstly, the roles of overexpressed sRNAs in biofilm formation, an important group behavior, were analyzed. Individual *E. coli* cells containing each sRNA-overexpressing plasmid were induced with 1 mM IPTG, and the extent of biofilm formation was measured. The extent of attached cells varied among experiments, depending on the incubation temperatures and times, *E. coli* strain tested, nutrient conditions, and surface material (data not shown). Since biofilm formation on round bottom polystyrene (PS) plates at 30^o^C was the most reproducible with minimal deviation, we analyzed the effects of individual sRNAs on biofilm formation under these conditions using the *E. coli* strain MG1655 grown on LB (Fig. 3a,b). Overexpression of Och5, CsrC, RseX, FnrS, SgrS, and GcvB induced >1.5-fold increase in biofilm formation, compared to control cells (containing the pHMB1 vector). In contrast, IS118, RyfD, SroC, SdsR, RyfA, McaS, OmrA, ArcZ, MicM, DicF, RybB, and DsrA led to a >1.5 fold decrease in biofilm development. Since sRNAs, such as Och5, GcvB, RyfA and DicF, caused slower cell growth upon overexpression (Fig. 3a,b), one possibility is that this cell growth inhibition is related to biofilm formation. However, these toxic sRNAs participated in inhibiting (RyfA, DicF) as well as enhancing biofilm formation (Och5, GcvB), suggesting no direct effects of growth inhibition on biofilm formation.

Hfq-dependent sRNAs usually require Hfq proteins for *in vivo* stability and function^16^,^18^. To determine whether Hfq protein is required for modulating biofilm formation by sRNAs, we examined the Hfq dependency of three selected inhibitory sRNAs (ArcZ, DsrA, and SdsR), previously identified as Hfq-associated sRNAs^30^,^35^,^36^, using *Δhfq* cells (Fig. 3c,d). Deletion of *hfq* induced a significant decrease in biofilm formation. Notably, overexpression of three sRNAs led to no further inhibition, suggesting that biofilm regulation by these sRNAs occurs through Hfq.

Moreover, ArcZ and DsrA, *rpoS*-activating sRNAs, inhibited biofilm formation, while RprA, another *rpoS*-activating sRNA, had little effect, indicating that inhibition of biofilm by the two former sRNAs is not directly related to RpoS.

We examined dosage effects of some sRNAs affecting biofilm formation to confirm that sRNAs are major contributors to this process. Since expression of sRNAs from the RNA expression vector is known to increase with the IPTG concentration^37^, the relationship between the IPTG and the phenotypic changes was analyzed. The activation or repression of biofilm formation also increased with the IPTG concentration (Supplementary Fig. 2), suggesting that biofilm phenotypes are proportional to sRNA concentrations.

### Small RNAs involved in swimming/swarming motility

**Figure 4:**
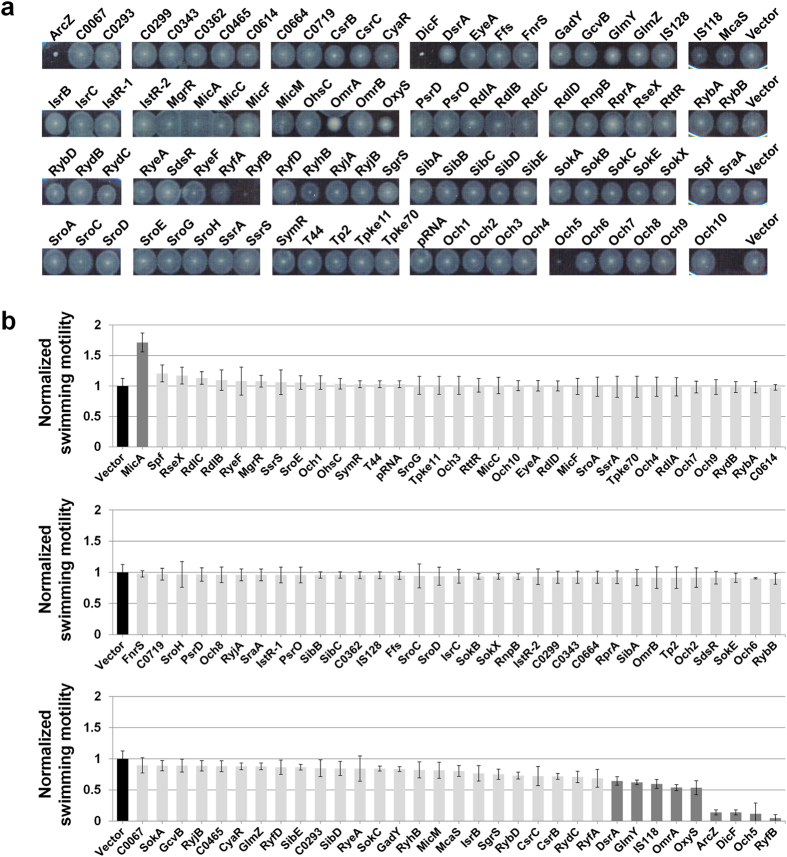
Effects of sRNA overexpression on swimming motility. (**a**) Swimming motility was investigated on soft agar plates (0.3% Bacto Agar, 1% tryptone, 0.5% NaCl) containing 1 mM IPTG and 100 μg/mL ampicillin. Assays were performed at 30 °C for 12 h. A representative image of at least three independent experimental sets is shown. (**b**) The diameter of the swimming circle was compared to that of the control strain harboring the pHMB1 vector. Results are presented as the average of at least three separate experiments and error bars represent standard deviation. Normalized motility of the control strain is indicated with a black bar, and strains displaying >1.5-fold changes with a dark grey bar.

**Figure 5:**
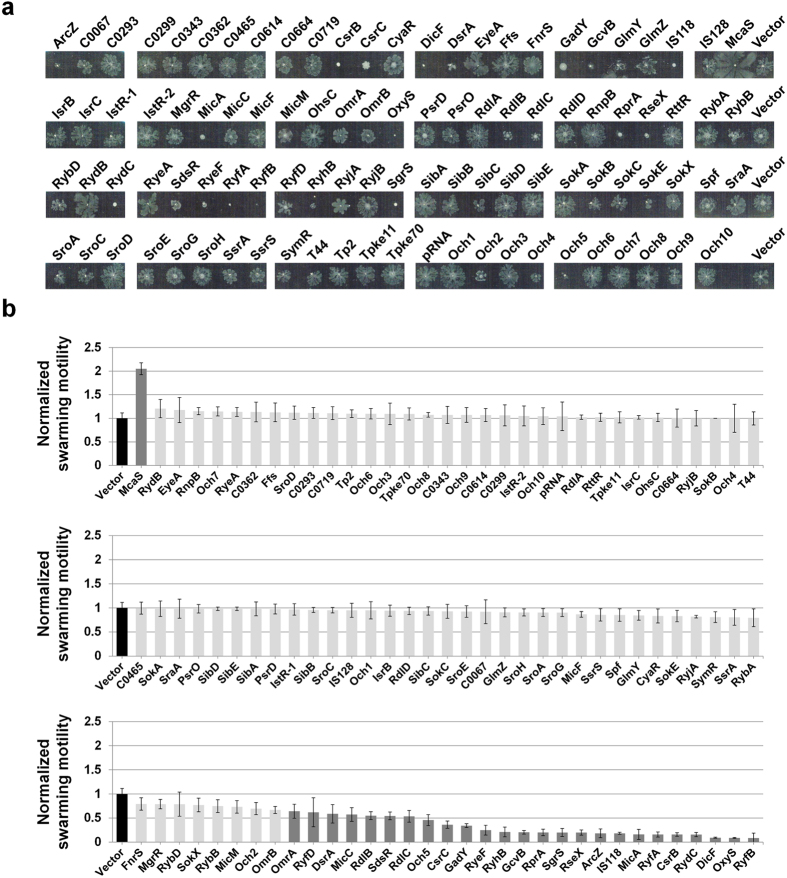
Effects of sRNA overexpression on swarming motility. (**a**) Swarming motility was investigated on soft agar plates (0.6% Eiken Agar, 0.5% glucose, 1% tryptone, 0.5% yeast extract, 0.5% NaCl) containing 1 mM IPTG and 100 μg/mL ampicillin. Overnight cultures of MG1655 cells harboring each sRNA-expressing plasmid were inoculated onto plates and incubated at 37 °C for 16 h. A representative image of at least three independent experimental sets is shown. (**b**) The distance of the swarming branch was measured and compared to the distance of control strain harboring the pHMB1 vector. Results are presented as the average of at least three separate experiments and the error bars represent standard deviation. Normalized motility of the control strain is indicated with a black bar, and strains displaying >1.5-fold changes with a dark grey bar.

Flagella-based motility is generally considered important for initial attachment, biofilm expansion, and dispersion of cells from surfaces of biofilm^20^,^26^,^38^,^39^. Two types of motility have been identified, depending on the bacterial flagella^40^. Swimming motility is an individual and random-directional movement in liquid medium, and essential for initial attachment to develop biofilm. Swarming motility is a coordinated bacterial social movement across the top of solid surface, accompanied by hyper-flagellation critical for surface colonization after initial attachment. Here, we further investigated whether these modes of motility are altered by individual sRNAs in correlation with biofilm formation. Overnight cultures of cells containing plasmids capable of overexpressing sRNAs upon IPTG induction were directly spotted on soft agar plates containing IPTG for assay of swimming or swarming motility. Nine sRNAs (DsrA, GlmY, IS118, OmrA, OxyS, ArcZ, DicF, Och5, and RyfB) induced a >1.5 fold decrease, while MicA promoted a >1.5 fold increase in swimming motility (Fig. 4). In terms of swarming motility, more sRNAs were effective (Fig. 5). In total, 25 sRNAs (OmrA, RyfD, DsrA, MicC, RdlB, SdsR, RdlC, Och5, CsrC, GadY, RyeF, RyhB, GcvB, RprA, SgrS, RseX, ArcZ, IS118, MicA, RyfA, CsrB, RydC, DicF, OxyS, and RyfB) reduced swarming motility by >1.5-fold (Fig. 5). Conversely, McaS induced a significant increase in swarming motility.

Among the nine sRNAs that suppressed swimming motility, DsrA, IS118, OmrA, OxyS, ArcZ, DicF, Och5 and RyfB also induced a significant reduction in swarming motility. On the other hand, GlmY led to severely reduced swimming motility, but mild reduction of swarming motility. SgrS, an sRNA suppressing glucose transport^41^, inhibited swarming motility significantly but swimming motility mildly, possibly attributable to inhibiting chemotaxis through repression of glucose transport. CsrB and CsrC, both of which antagonize CsrA protein, a carbon storage regulator, also caused a severe reduction in swarming motility and mild reduction in swimming motility, probably through inhibition of glucose metabolism. Other sRNAs that reduced swarming motility severely and swimming motility mildly were RprA, SdsR, GcvB, RyfD, GadY, RyhB, and RyfA. Our results indicate that swimming and swarming motility mechanisms share common pathways.

Notably, however, MicA sRNA, a post-transcriptional regulator of the outer membrane protein, OmpA^42^,^43^, increased swimming motility but almost eliminated swarming motility. In contrast, overexpression of McaS led to increased swarming motility but reduced swimming motility, suggesting that MicA and McaS affect their target genes independently of each other, and their control levels are possibly located downstream of the common pathways.

### Small RNAs involved in type I and curli fimbriae formation

In addition to flagella-based swim/swarm motility, cell surface appendages, such as type I fimbriae and curli fimbriae, are implicated in biofilm formation^20^,^26^,^44^,^45^. Type I fimbriae are rod-shape adhesive organelles surrounding the cell surface, thought to be critical for initial stable, irreversible attachment in biofilm development. Since mannose-specific adhesin, FimH, appears at the tip of type I fimbriae, expression of these organelles could be indirectly determined via examining agglutination of mannose-rich yeast cells^46^,^47^. Curli fimbriae are also adhesive protein appendages that play a role in cell-surface and cell-cell interactions. Formation of curli fimbriae is thought to be essential for development of mature biofilm. The level of curli expression can be monitored by the red color of colonies on Congo red indicator plates^44^. Specifically, cells expressing curli fimbriae appear as red colonies on plates stained with Congo red, whereas those lacking curli fimbriae remain as white colonies. In the current study, we examined the effects of sRNA overexpression on biosynthesis of both type I and curli fimbriae and how these phenotypic changes are related to biofilm formation.

**Figure 6:**
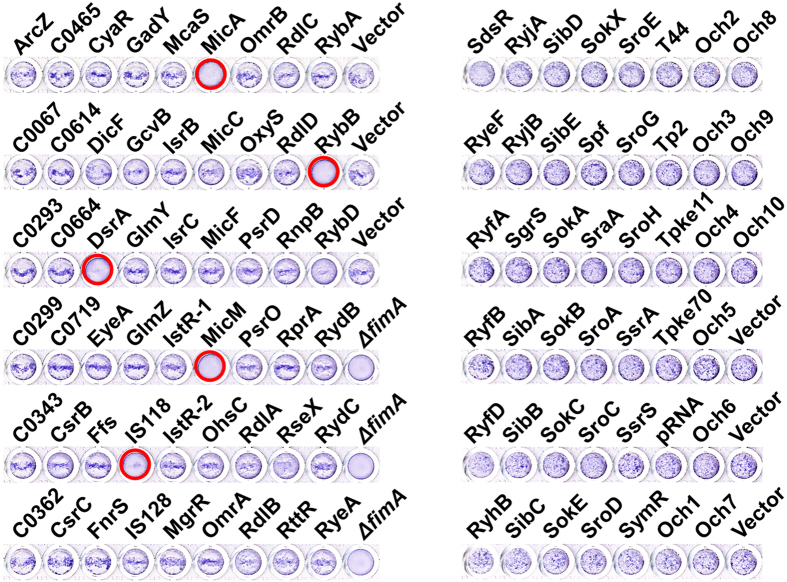
Effects of sRNA overexpression on type I fimbriae phenotypes. Formation of mannose-specific type I fimbriae was determined by the ability of each strain to agglutinate yeast cells. Strains were grown in LB containing 1 mM IPTG and 100 μg/mL ampicillin without shaking at 37^o^C and mixed with the same volume of yeast suspension (0.5% w/v, PBS) in a 96-well titer plate. Crystal violet was added to enhance observation. The data are representative of at least three independent experiments. The *ΔfimA* strain was used as a type I fimbriae-deficient control. Strains that appeared type I fimbriae-deficient are indicated with circles.

MG1655 cells overexpressing DsrA, IS118, MicA, MicM, and RybB appeared severely deficient in type I fimbriae synthesis (Fig. 6) in correlation with their ability to inhibit biofilm formation (Fig. 3). Transcription of MicA and RybB is dependent on sigma factor σ^E^, which is activated by cell envelope stress, and these sRNAs regulate mRNAs of distinct and shared targets^48^. In particular, RybB and MicA suppress expression of *fimA* encoding a major subunit of type I fimbriae and *fimB* encoding recombinase required for *fimA* promoter inversion, respectively^49^. However, no involvement of DsrA, IS118, and MicM in type I fimbriae has been reported to date.

**Figure 7:**
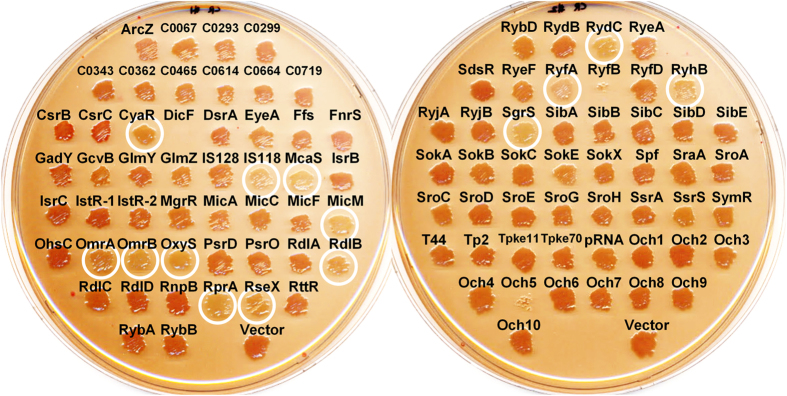
Effects of sRNA overexpression on curli fimbriae phenotype. Strains harboring the sRNA library were assayed on a Congo red agar plate (LB agar without NaCl containing 1 mM IPTG, 100 μg/mL ampicillin, 40 μg/mL Congo red, 20 μg/mL Coomassie blue) to evaluate expression of curli fimbriae. Overnight cultures were streaked on agar plates and grown at 28 °C for 48 h. Strains showing curli fimbriae deficiency are indicated with white circles.

Curli synthesis was affected by a variety of sRNAs (Fig. 7). MG1655 cells overexpressing CyaR, IS118, McaS, MicM, OmrA, OmrB, OxyS, RdlB, RprA, RseX, RydC, RyfA, RyhB, and SgrS appeared curli-deficient. The major regulator of curli fimbriae, CsgD, was recently identified as a gene negatively regulated by several sRNAs, including McaS, OmrA, OmrB, RprA, RydC, and GcvB^50^,^51^. Our findings are consistent with these results, with the exception of GcvB, which did not affect curli expression in our experiments. Overexpression of CsrB or CsrC, both inactivators of CsrA, resulted in a more reddish color on the Congo red indicator plate.

### Effects of sRNA overexpression on biofilm-related genes

**Figure 8:**
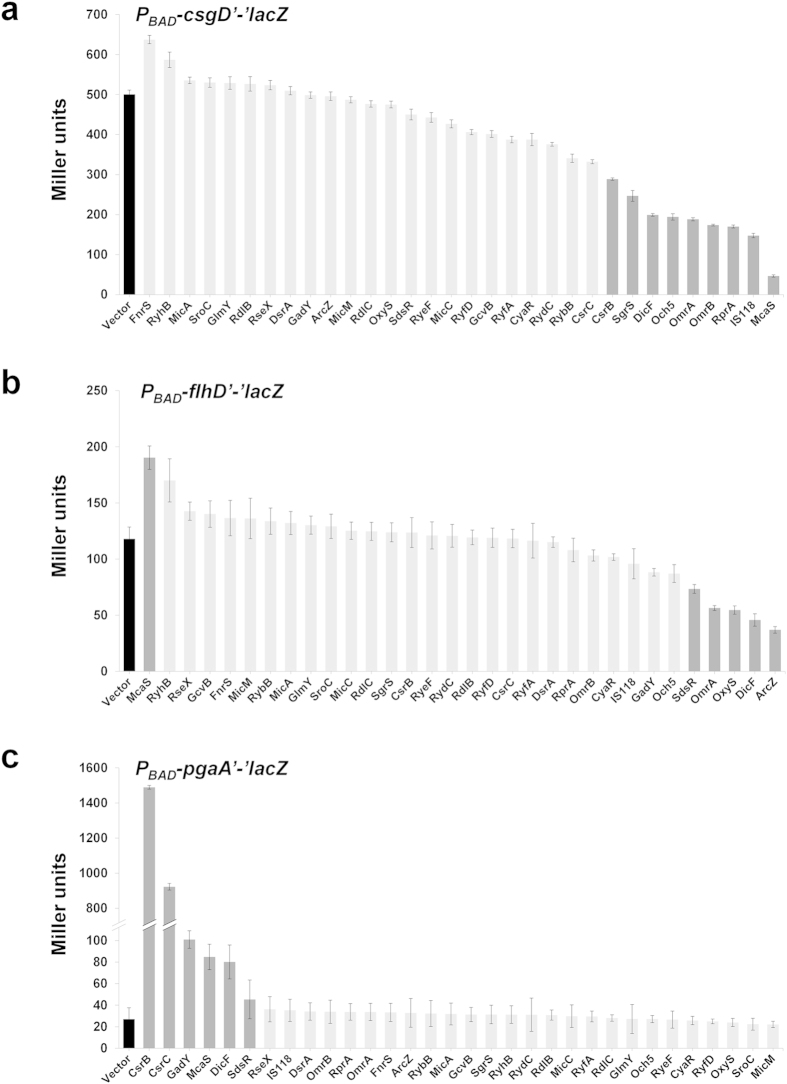
Effects of overexpressed sRNAs on expression of *csgD’-, flhD’-,* and *pgaA’-‘lacZ* translational fusions. *E. coli* GSO559 (P_BAD_-*csgD’-‘lacZ)* (**a**), GSO563 (P_BAD_-*flhD’-‘lacZ*) (**b**), and GSO567 (P_BAD_-*pgaA’-‘lacZ* ) (**c**) were treated with arabinose and IPTG. β-galactosidase activities were measured after the arabinose and IPTG induction. Cells overexpressing RyfB were not used for β-galactosidase due to their severe growth defect. Results are presented as the average of at least three independent experiments and error bars correspond to standard deviation. The control strains carrying the vector are indicated with a black bar, and strains displaying >1.5-fold changes with a dark grey bar.

Effects of overexpression of the 33 sRNAs on expression of three biofilm-related genes, *csgD, flhD* and *pgaA* were examined using each *lacZ* fusion. The transcriptional regulator CsgD is known to be a key player in the regulatory circuit for biofilm formation^52^. Besides OmrA, OmrB, RprA and McaS that were previously reported as negative regulators of *csgD*^33^,^50^,^53^,^54^, CsrB, DicF, IS118, Och5 and SgrS reduced the expression of the *csgD-lacZ* translational fusion by >1.5 fold (Fig. 8a). All *csgD*-repressing sRNAs except Och5, DicF, and CsrB decreased curli fimbriae formation. As for Och5 and DicF, we were unable to evaluate their effects on curli fimbriae formation because of poor growth of cells on assay conditions for curli expression (Fig. 7). CsrB, which showed a moderate repression of *csgD* expression, caused a more reddish color on Congo red plates (Fig. 7). Since, however, other *csgD*-unlinked sRNAs, such as OxyS and RseX, also repressed curli formation (Fig. 7), curli-deficiency can occur without suppression of *csgD* expression. Och5 and SgrS caused an increase in biofilm formation, while DicF and IS118 inhibited biofilm development (Fig. 3), suggesting that *csgD* expression alone does not explain biofilm formation. We analyzed possible interactions between newly identified *csgD*-repressing sRNAs (DicF, IS118, Och5 and SgrS) and *csgD* mRNA by using electrophoretic mobility shift assay (EMSA) (Supplementary Fig. 3). Only Och5 formed a complex with the *csgD* 5′-UTR, suggesting that it negatively regulate *csgD* expression by direct base-pairing with the 5′-UTR. The other sRNAs DicF, IS118 and SgrS may not directly target the *csgD* 5′-UTR. We also confirmed that RprA, OmrA and OrmB can form complexes with the *csgD* 5′-UTR *in vitro*.

Transcriptional regulator FlhDC can be involved in biofilm formation by playing a key role in the flagellar assembly process^55^. We found that expression of *flhD-lacZ* was repressed upon overexpression of OmrA, ArcZ, OxyS, SdsR, and DicF by >1.5-fold (Fig. 8b). Involvement of DicF in down-regulation of *flhD* expression has not been reported to date^31^,^54^. All these sRNAs showed a reduced swarming phenotype, while McaS that activates *flhD* expression caused an increase in swarming motility, suggesting that *flhD* expression is closely related to the swarming phenotype. In addition, overexpression of OmrA, ArcZ, DicF, and SdsR, but not OxyS, led to a reduction in biofilm formation (Fig. 3). We also identified McaS as activating *flhD-lacZ* expression. McaS has been known as a positive regulator of *flhD*^54^. Possible interaction of newly found *flhD*-repressing sRNA DicF with *flhD* mRNA was also analyzed using EMSA (Supplementary Fig. 3). We did not detect any DicF-*flhD* 5′-UTR complexes, suggesting that the *flhD* 5′-UTR may not be the direct target mRNA of DicF. Interestingly, we did not find complexes between the *flhD* 5′-UTR and OmrA or OmrB either, even though De Lay and Gottesman^31^ suggested their binding to the *flhD* 5′-UTR through mutational analysis. No interaction of SdsR was observed either, which could be predicted from the previous mutagenesis data^31^.

The *pgaABCD* operon encodes the enzymes and porin responsible for synthesis of a biofilm polysaccharide adhesion^56^. We found that CsrB, CsrC, DicF, McaS, GadY and SdsR activated *pgaA-lacZ* expression by >1.5 fold (Fig. 8c). In addition to previously reported McaS, CsrB and CsrC^57^,^58^,^59^, we identified GadY, DicF and SdsR as new positive regulators of *pgaA*. However, only CsrC (and CsrB) increased biofilm formation.

### Biofilm formation or related phenotypes in strains lacking sRNAs

**Figure 9:**
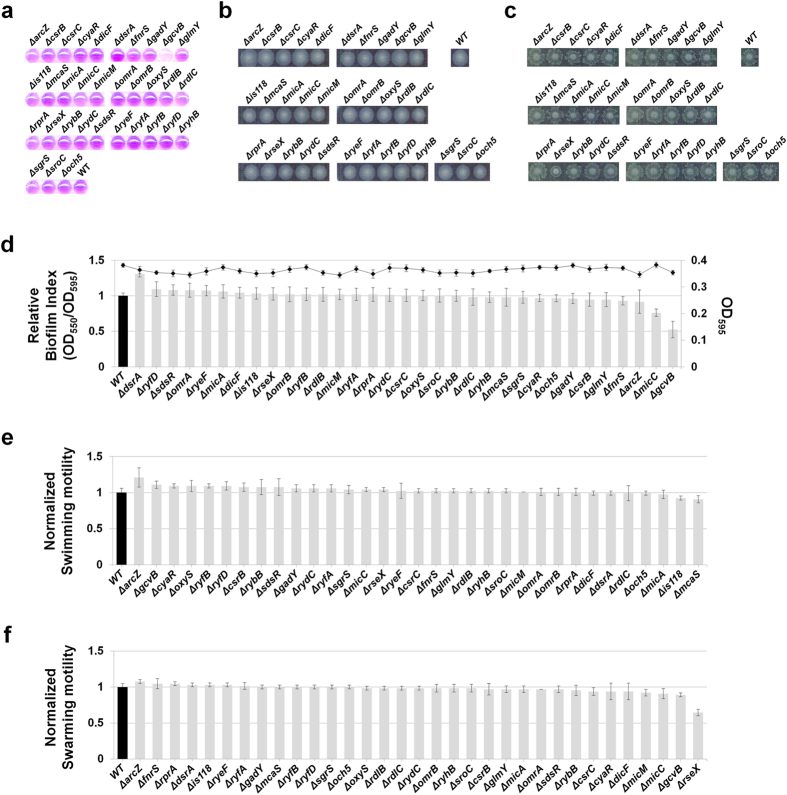
Biofilm formation and swimming/swarming motility in strains lacking sRNAs. Biofilm formation and swimming/swarming motility were analyzed in strains lacking each of 33 sRNAs, which were shown to significantly affect biofilm formation and related phenotypes. sRNA knock-out strains containing the RNA expression vector were tested under the same experimental conditions employed with sRNA-overexpressing cells. Biofilm formation (**a**,**d**), and swimming (**b**,**e**) and swarming motility (**c**,**f**).

**Figure 10:**
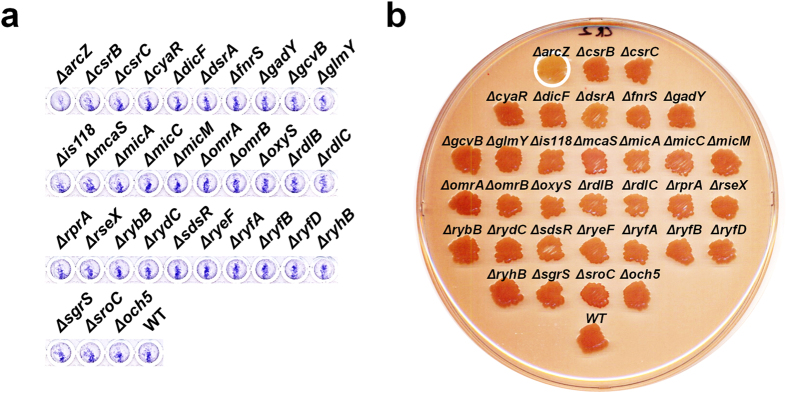
Type I fimbriae and curli fimbriae formation in strains lacking sRNAs. sRNA knock-out strains containing the RNA expression vector were tested under the same experimental conditions employed with sRNA-overexpressing cells. Type I fimbriae (**a**) and curli fimbriae (**b**).

We also examined biofilm formation or related phenotypes in mutant strains lacking sRNAs. We tested the thirty-three sRNAs that were shown to affect these cellular processes upon overexpression. sRNA knock-out strains containing the RNA expression vector were tested under the same experimental conditions employed with sRNA-overexpressing cells. MG1655*∆dsrA*, a mutant strain lacking DsrA, increased biofilm formation, while MG1655*∆gcvB* decreased biofilm formation (Fig. 9a,d). MG1655*∆arcZ* displayed a slight increase in swimming motility (Fig. 9b,e). These phenotypes were opposite to the overexpression phenotypes, suggesting that the chromosomally-expressed sRNAs give the same phenotypes in our experimental conditions. MG1655*∆micC* showed a slightly reduced biofilm formation, suggesting that MicC is also involved in inducing biofilm formation although its overexpression causes no further increase of biofilm formation (Fig. 3). MG1655*∆rseX* showed slightly reduced swarming motility (Fig. 9c,f). As overexpression of RseX also suppressed swarming motility (Fig. 5), it seems likely that proper amounts of RseX are needed for efficient swarming motility. It was intriguing that only for the few cases the deletion of sRNA genes had effects on biofilm formation or motility phenotypes. In addition, we did not find any sRNA showing significant changes in type I and curli fimbriae formation except for the *arcZ* knock-out that showed a slightly reduced type I fimbriae formation and reduction of curli fimbriae (Fig. 10). We reason that some sRNAs may not be expressed enough to affect these cellular processes in our experimental conditions or there might be redundancy of sRNAs acting on the processes.

## Discussion

**Figure 11:**
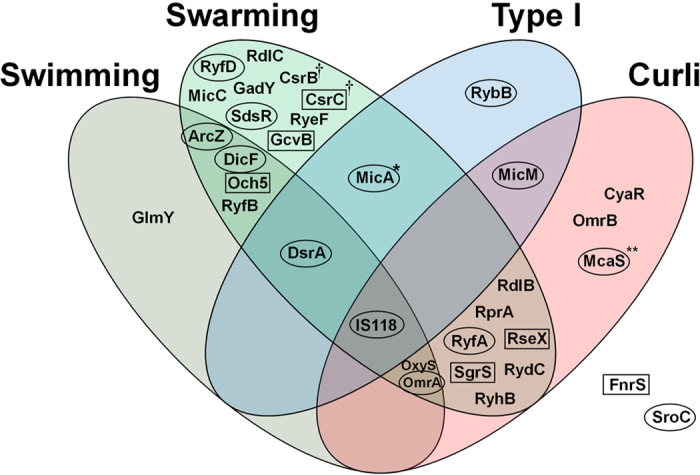
Venn diagram of sRNAs differentially affecting biofilm formation and related phenotypes. sRNAs that increase and decrease biofilm formation >1.5-fold are shown in boxes and ellipses, respectively. All sRNAs except for MicA and McaS in the Venn diagram inhibit one or more biofilm-related phenotypes. MicA that increases swimming motility >1.5-fold is marked ‘*’, McaS that increases swarming motility >1.5-fold ‘**’, and CsrB and CsrC that generate more reddish color on Congo red plates ‘†’. FnrS and SroC shown outside the Venn diagram affected biofilm formation positively and negatively, respectively, without affecting the biofilm-related phenotypes.

In the current study, a collection of plasmids overexpressing 99 experimentally validated *E. coli* sRNAs was constructed and used to examine the functions of individual sRNAs in biofilm formation and associated phenotypes. Thirty-three of the sRNAs affected biofilm formation, swimming or swarming motility and formation of type I and curli fimbria by >1.5-fold (Table 1 and Fig. 11). However, it should be noted that the observed phenotypes would be the cumulative results of direct and/or indirect effects caused by long-term induction of sRNAs.

Among the 18 sRNAs affecting biofilm formation, 12 caused a decrease while 6 induced an increase. Notably, swarming was decreased by 25 sRNAs but increased by McaS only, while swimming was suppressed by 9 sRNAs and increased by MicA only. Curli formation was suppressed by 14 sRNAs but activated by CsrB and CsrC. Interestingly, CsrB and CsrC promoted both biofilm and curli formation. Conversely, however, RseX and SgrS, other sRNAs that enhanced biofilm formation, repressed curli formation. The remaining biofilm-promoting sRNAs, Och5 and GcvB, suppressed swimming and swarming, respectively. MicA, which induced an increase in swimming, suppressed biofilm formation only slightly, but both swarming motility and type I fimbriae formation to a significant extent. McaS, which caused an increase in swarming, severely inhibited both biofilm and curli fimbriae formation. Type I fimbriae formation was suppressed by 5 sRNAs, all of which also inhibited biofilm formation, suggesting that these processes are closely related and that cell attachment plays an important role in the development of biofilm. While the involvement of MicA and RybB in type I fimbriae formation has been previously documented, DsrA, IS118, and MicM sRNAs were identified for the first time in this study. Except for type I fimbriae formation, biofilm development was not related to repression of any of the four phenotypes. The majority of sRNAs suppressed swarming motility and curli fimbriae. Interestingly, IS118, which is yet to be characterized, suppressed all phenotypes, including biofilm formation. FnrS and SroC affected only biofilm formation positively and negatively, respectively. CyaR and OmrB specifically suppressed curli fimbriae formation, RybB type I fimbriae formation, and GlmY swimming. Several sRNAs (RyfD, RdlC, MicC, GadY, SdsR, RyeF, and GcvB) repressed swarming only. While most sRNAs effective in biofilm formation, motility, and/or fimbriae formation have been identified as Hfq-dependent, some Hfq-independent sRNAs, such as CsrB, CsrC, RdlB, RdlC, RyfA, RyfB, RyfD and Och5, are also involved in these processes.

Involvement of sRNAs in motility and biofilm formation has been documented recently. Limited sRNAs are reported to directly affect biofilm formation and motility. ArcZ sRNA is a major regulator of rdar (red, dry, and rough) biofilm development in *Salmonella*^60^. Our experiments showed that overexpression of ArcZ in *E. coli* leads to suppression of swimming and swarming motility as well as biofilm formation. McaS acts as a positive regulator of biofilm formation by activating *pgaABCD* operon^54^, the cell-bound exopolysaccharide adhesin. In our study, McaS increased swarming motility, but repressed biofilm and curli fimbriae formation. The reasons underlying this discrepancy with regard to effects on biofilm formation remain to be established. Besides previously known McaS, CsrB and CsrC^57^,^58^,^59^, we identified three more sRNAs, DicF, GadY and SdsR, as positive regulators of *pgaA*. Since CsrB and CsrC enhanced biofilm formation and much highly activated *pgaA* expression compared to the other sRNAs, *pgaA* expression more than threshold levels seems to be required for induction of biofilm formation. Previously, De Lay and Gottesman used a collection of plasmids overexpressing 26 Hfq-dependent sRNAs to perform phenotypic screening of swimming motility^31^. The group found that ArcZ, OmrA, OmrB and OxyS repress swimming motility, while McaS increases swimming motility. Moreover, this regulation occurs through *flhDC*. Their experiments additionally showed that MicA increases swimming motility in a manner not associated with *flhDC*. In our study, ArcZ, OmrA, OxyS and DicF suppressed swimming motility with down-regulation of *flhD*, while OmrB and McaS, which showed null and positive effects on *flhD* expression, respectively, had no significant effects on swimming motility. SdsR that moderately down-regulated *flhD* expression displayed little change in swimming motility. This discrepancy may be attributed to differences in the genetic backgrounds of strains used by the two groups. Since some stocks of strain MG1655 are reported to have the IS element inserted upstream of the *flhDC* operon^61^, which could activate the *flhDC* promoter and increase motility, we checked our laboratory stock of this strain. The results showed that the IS1 element was transposed in the *flhDC* promoter region (Supplementary Fig. 4). However, the reason for significant enhancement of swarming motility by McaS sRNA remains to be determined.

The impact of some sRNAs on biofilm formation can be explained in terms of their known functions. CsrB and CsrC are antagonists of CsrA protein, and *E. coli* cells lacking CsrA are reported to increase biofilm formation and activate biofilm disposal^62^. We found that overexpression of CsrB and CsrC led to increased biofilm and curli formation, but inhibited swarming motility. Since inhibition of swarming motility is not related to biofilm formation (Fig. 8), CsrA may be involved in linking the pathways of biofilm formation and curli synthesis.

Appropriate levels of RpoS are important for normal biofilm development. RpoS is differentially expressed during biofilm development^63^ and its absence or elevated levels leads to inhibition of biofilm formation^64^,^65^. Among three *rpoS*-activating sRNAs, ArcZ and DsrA severely inhibited biofilm formation, while repression by RprA was mild. Previously, we showed that overexpression of the three *rpoS*-activating sRNAs induces comparable levels of *rpoS* translation^66^. Therefore, the amount of RpoS is not critical, but other effects of the inducing signals appear important. In addition, the three *rpoS*-activating sRNAs differentially affected the four biofilm-related phenotypes. Specifically, ArcZ and DsrA repressed swimming and swarming motilities, DsrA additionally inhibited type I fimbriae formation, and RprA suppressed swarming motility and curli fimbriae formation.

Jorgensen *et al.*^67^ reported that McaS, RprA and GcvB inhibit *csgD* translation through direct base-pairing. OmrA and OmrB have also been shown to repress *csgD* translation^33^. Although CsgD is a curli biosynthesis regulator, the effects of these sRNAs on curli formation were different. McaS, RprA, OmrA, and OmrB inhibited curli formation, but not GcvB. Furthermore, CsrB, DicF, IS118, Och5 and SgrS we identified as additional repressors of *csgD* also showed different phenotypes of curli formation. The finding that suppression of *csgD* expression is not sufficient to inhibit curli formation suggests the presence of another regulatory step.

We have shown that except for the few sRNAs the sRNA gene knock-out had little effects on biofilm formation or related phenotypes. Deletion of only *dsrA, gcvB,* or *arcZ* showed opposite phenotypes to those upon overexpression of the corresponding sRNAs. We reason that some sRNAs may not be expressed enough to affect the processes in our experimental conditions or there might be redundancy of sRNAs acting on the processes. Nevertheless, we found additional effects of sRNA gene knock-out, such as a slight reduction of biofilm formation by *micC* knock-out and a slight reduction of swarming by *rseX* knock-out, which could not be expected from the phenotypes upon overexpression.

Biofilm development should be regulated precisely and dynamically according to time- or environment-specific requirements. sRNAs appear to regulate biofilm formation in distinct ways that are not fully co-related to motility and type I or curli fimbriae phenotypes, reflecting the complexity of the regulatory pathways involved in development of biofilm. It is possible that these sRNAs act via different mechanisms to link various environmental signals and the decision process for biofilm formation. Further detailed analyses are warranted to elucidate the roles of individual sRNAs in biofilm development.

## Materials and Methods

### Bacterial strains, plasmids, and oligonucleotides

*E. coli* K-12 MG1655 strain was used for phenotypic analyses. For construction and propagation of plasmids, the *E. coli* strain, DH5α, was used. The *hfq* mutant strain was obtained from *E. coli* Keio strain collection^68^. *E. coli* strains GSO559, GSO563, and GSO567 carried *csgD*-, *flhD*-, and *pgaA-lacZ* translational fusions, respectively^54^. For IPTG-inducible stable overexpression of each sRNA, the RNA expression vectors, pHM-tac^69^, pHMB1, and pHMB2, were employed. The pHMB1 plasmid was constructed by adding the modified *rnpB* terminator sequence^70^ to the region downstream of the *Hin*dIII cloning site of pHM-tac^66^. Plasmid pHMB2 was constructed by replacing the *Eco*RI cloning site of pHMB1 with *Sma*I for blunt-end ligation. The oligonucleotide sequences used for plasmid construction are listed in Supplementary Table 3. Plasmids pKD13 and pKD46 were used for λ Red-mediated recombination to generate sRNA knock-out strains^71^.

### Construction of the sRNA expression library

To generate a collection of sRNA-expressing plasmids, each sRNA coding region was amplified from MG1655 genomic DNA via PCR with the primers described in Supplementary Table 3. sRNA genes were amplified so that they could be transcribed from their natural transcription +1 site and terminated either at their own transcriptional termination site or the modified *rnpB* terminator encoded within the vector sequence. In cases where transcription units were not fully known, 20 nt upstream chromosomal sequences were added to the predicted 5′ ends. To ensure termination of transcription, >25 nt downstream chromosomal sequences were added to the predicted 3′ ends or the identified ρ-independent terminators. For more efficient transcriptional initiation, an additional adenine nucleotide was incorporated, if necessary, to ensure that the start site for sRNAs was adenine. PCR products were digested with *Eco*RI/*Xba*I, *Sma*I/*Xba*I or *Eco*RI/*Hin*dIII, followed by ligation into pHM-tac, pHMB1, or pHMB2 plasmids. Ligation products were transformed into strain DH5α, and selected on ampicillin-containing LB plates for propagation. For sRNAs with highly homologous sequences or less than 150 nt in length, coding DNAs were obtained from a direct gene synthesis service (Bioneer).

### Construction of sRNA knock-out strains

sRNA knock-out strains were constructed in strain MG1655 using λ Red-mediated recombination with PCR fragments containing both the upstream 55 bp and the downstream 55 bp region of each sRNA gene, as previously described^71^. The kanamycin resistance cassette was inserted in the opposite direction of sRNA expression. To avoid disruption of the surrounding genes, the overlapping or nearby gene sequences were kept unchanged. In case of SgrS and SdsR, the entire sRNA sequences were deleted although their genes are overlapped with *sgrT* and *ryeA,* respectively. The replacement of each sRNA gene with the kanamycin resistance cassette was confirmed by PCR and sequencing analysis. Oligonucleotides used to generate sRNA knock-out strains are listed in in Supplementary Table S3.

### RNA extraction and northern blot analysis

Cells were grown overnight in LB broth with 100 μg/mL ampicillin, where necessary. Overnight cultures were diluted 1:100 in fresh medium, and growth continued to the exponential phase (OD_600_ ≈ 0.6). IPTG (1 mM) was added to cultures for sRNA overexpression. After 20 min, cultures were directly extracted using the acidic hot phenol method, as described previously^70^.

Northern blot analysis was performed as follows: 10 μg total RNAs were fractionated on a 5% polyacrylamide gel containing 7 M urea, and electrotransferred onto a Hybond^TM^-XL membrane (Amersham Biosciences) via TE70 ECL Semi-dry transfer unit (Amersham Biosciences) at 185 mA for 1 h. Membranes were hybridized with 5′-^32^P labeled oligonucleotide probes in Rapid-Hyb buffer (Amersham Biosciences) at 42 °C, according to the manufacturer’s instructions. In most cases, the probe rnpBXb1 was used for detection of overexpressed sRNA from library plasmids. In cases where sRNA was not detected with rnpBXb1, specific oligonucleotide probes were used (listed in Supplementary Table 3). Hybridization signals were assessed using Image Analyzer FLA7000 (Fuji).

### Electrophoretic mobility shift assay

EMSA experiments were performed as previously described^72^. 5′-UTR regions of *csgD* and *flhD* and coding regions of sRNAs were amplified using PCR with primers containing the T7 promoter sequence. Oligonucleotides used for PCR amplification are listed in in Supplementary Table S3. RNAs were prepared by *in vitro* transcription using T7 RNA polymerase (Promega), with the PCR fragments as templates. 5′ end-labeled the *csgD* or *flhD* UTR (5 nM) was incubated with unlabeled sRNAs (0.5 or 2.5 μM) in 10 μl TMN buffer [100 mM Tris-acetate, pH 7.6, 500 mM NaOAc, 25 mM Mg(OAc)_2_] at 25 °C for 20 min. The reactions were then analyzed on a 5% (v/v) non-denaturing polyacrylamide gel at 4 °C.

### Assay for biofilm formation

Overnight cultures of MG1655 cells containing individual sRNA overexpression plasmids were diluted 100-fold into 100 μL of fresh LB medium in 96-well microtiter plates (polystyrene; SPL Lifesciences Cat. 34296) containing 1 mM IPTG and 100 μg/mL ampicillin, and cultured without shaking for 12 h at 30 °C. The amount of biofilm attached to the microtiter plate was measured via crystal violet staining. Specifically, after discarding cell cultures, plates were washed twice by submerging in distilled water and shaking out, followed by the addition of 125 μL 0.1% (w/v) crystal violet water solution to each well. After 10 min incubation for staining, plates were washed four times with distilled water. Next, 30% (v/v) acetic acid solution was added to solubilize stained crystal violet, and OD_550_ estimated with the Emax microplate reader (Molecular Devices). OD_550_ values were divided by OD_595_ values of cell density to normalize all values per OD_595_ unit, and termed ‘biofilm index’.

### Assay for swimming/swarming motility

Swimming and swarming motilities were investigated on soft agar plates. A 1 μL aliquot of each overnight culture was inoculated using a pipette tip onto swim agar (0.3% Bacto Agar, 1% tryptone, 0.5% NaCl) or swarm agar (0.6% Eiken Agar, 0.5% glucose, 1% tryptone, 0.5% yeast extract, 0.5% NaCl) containing 1 mM IPTG and 100 μg/mL ampicillin. Agar plates were sealed with Saran Wrap to prevent dehydration. The swimming assay was performed at 30 °C for 12 h, and swarming assay at 37 °C for 16 h. The diameter of the swimming circle or distance of swarming branch was measured and normalized against that of a control strain.

### Assay for type I fimbriae expression

Mannose-specific type I fimbriae expression was monitored using the yeast agglutination assay. Stagnant overnight cultures of each strain and the same volume of 0.5% (w/v) yeast (Sigma, YSC2) suspension in PBS were mixed, and aggregation estimated after 20 min incubation at room temperature by visual inspection. A 100th volume of 0.1% crystal violet solution was also added for enhanced observation.

### Assay for curli expression

Production of curli fimbriae was determined based on uptake of red color on Congo red plates (LB agar without NaCl containing 1 mM IPTG, 100 μg/mL ampicillin, 40 μg/mL Congo red and 20 μg/mL Coomassie Blue). MG1655 cells harboring each sRNA overexpression plasmid were streaked onto Congo red plates, and incubated for 48 h at 28 °C. Colonies displaying pink-white color were considered curli fimbriae-deficient.

### β-galactosidase assay

Overnight cultures grown in LB were diluted 100-fold into fresh LB containing ampicillin (100 μg/mL) and grown at 37 °C for 1.5 h. The *lacZ* fused target gene was induced by adding 0.2% arabinose and sRNA expression was simultaneously induced by 1 mM IPTG. After 1.5-h induction, β-galactosidase activities were determined, as described previously^73^.

## Additional Information

**How to cite this article**: Bak, G. *et al.* Identification of novel sRNAs involved in biofilm formation, motility, and fimbriae formation in *Escherichia coli. Sci. Rep.* **5**, 15287; doi: 10.1038/srep15287 (2015).

## Supplementary Material

Supplementary Information

## Figures and Tables

**Table 1:** Effects of *E. coli* sRNAs on biofilm phenotypes upon overexpression.

**sRNA**	**Relative Biofilm**^a^	**Motility**^b^		**Fimbriae**	
		**Swimming**	**Swarming**	**Type I**^c^	**Curli**^d^
Vector	100 ± 6%	—	—	—	—
ArcZ	18 ± 4%	↓	↓	—	—
C0067	91 ± 17%	—	—	—	—
C0293	83 ± 10%	—	—	—	—
C0299	81 ± 14%	—	—	—	—
C0343	73 ± 7%	—	—	—	—
C0362	78 ± 16%	—	—	—	—
C0465	90 ± 17%	—	—	—	—
C0614	87 ± 15%	—	—	—	—
C0664	99 ± 17%	—	—	—	—
C0719	99 ± 15%	—	—	—	—
CsrB	145 ± 13%	—	↓	—	↑
CsrC	182 ± 24%	—	↓	—	↑
CyaR	80 ± 16%	—	—	—	↓
DicF	11 ± 5%	↓	↓	—	ND^e^
DsrA	2 ± 1%	↓	↓	↓	—
EyeA	100 ± 10%	—	—	—	—
Ffs	90 ± 12%	—	—	—	—
FnrS	165 ± 5%	—	—	—	—
GadY	116 ± 13%	—	↓	—	—
GcvB	150 ± 10%	—	↓	—	—
GlmY	122 ± 18%	↓	—	—	—
GlmZ	139 ± 10%	—	—	—	—
IS118	66 ± 10%	↓	↓	↓	↓
IS128	83 ± 12%	—	—	—	—
McaS	53 ± 16%	—	↑	—	↓
IsrB	100 ± 13%	—	—	—	—
IsrC	97 ± 12%	—	—	—	—
IstR-1	105 ± 12%	—	—	—	—
IstR-2	105 ± 13%	—	—	—	—
MgrR	120 ± 5%	—	—	—	—
MicA	71 ± 6%	↑	↓	↓	—
MicC	104 ± 11%	—	↓	—	—
MicF	96 ± 10%	—	—	—	—
MicM	12 ± 2%	—	—	↓	↓
OhsC	107 ± 8%	—	—	—	—
OmrA	44 ± 4%	↓	↓	—	↓
OmrB	108 ± 3%	—	—	—	↓
OxyS	107 ± 3%	↓	↓	—	↓
PsrD	109 ± 9%	—	—	—	—
PsrO	105 ± 9%	—	—	—	—
RdlA	103 ± 8%	—	—	—	—
RdlB	106 ± 6%	—	↓	—	↓
RdlC	103 ± 2%	—	↓	—	—
RdlD	105 ± 2%	—	—	—	—
RnpB	114 ± 2%	—	—	—	—
RprA	82 ± 3%	—	↓	—	↓
RseX	174 ± 12%	—	↓	—	↓
RttR	97 ± 9%	—	—	—	—
RybA	112 ± 13%	—	—	—	—
RybB	10 ± 2%	—	—	↓	—
RybD	113 ± 10%	—	—	—	—
RydB	114 ± 3%	—	—	—	—
RydC	99 ± 5%	—	↓	—	↓
RyeA	108 ± 4%	—	—	—	—
SdsR	57 ± 11%	—	↓	—	—
RyeF	119 ± 10%	—	↓	—	—
RyfA	56 ± 22%	—	↓	—	↓
RyfB	127 ± 2%	↓	↓	—	ND
RyfD	66 ± 13%	—	↓	—	—
RyhB	92 ± 11%	—	↓	—	↓
RyjA	88 ± 10%	—	—	—	—
RyjB	94 ± 15%	—	—	—	—
SgrS	160 ± 18%	—	↓	—	↓
SibA	99 ± 13%	—	—	—	—
SibB	88 ± 15%	—	—	—	—
SibC	86 ± 14%	—	—	—	—
SibD	99 ± 11%	—	—	—	—
SibE	104 ± 14%	—	—	—	—
SokA	101 ± 14%	—	—	—	—
SokB	107 ± 14%	—	—	—	—
SokC	87 ± 14%	—	—	—	—
SokE	96 ± 11%	—	—	—	—
SokX	87 ± 7%	—	—	—	—
Spf	110 ± 12%	—	—	—	—
SraA	122 ± 13%	—	—	—	—
SroA	96 ± 13%	—	—	—	—
SroC	63 ± 6%	—	—	—	—
SroD	87 ± 14%	—	—	—	—
SroE	98 ± 10%	—	—	—	—
SroG	115 ± 10%	—	—	—	—
SroH	112 ± 16%	—	—	—	—
SsrA	120 ± 8%	—	—	—	—
SsrS	134 ± 9%	—	—	—	—
SymR	98 ± 9%	—	—	—	—
T44	106 ± 7%	—	—	—	—
Tp2	109 ± 8%	—	—	—	—
Tpke11	120 ± 7%	—	—	—	—
Tpke70	119 ± 10%	—	—	—	—
pRNA	118 ± 8%	—	—	—	—
Och1	109 ± 9%	—	—	—	—
Och2	92 ± 10%	—	—	—	—
Och3	107 ± 10%	—	—	—	—
Och4	99 ± 11%	—	—	—	—
Och5	229 ± 10%	↓	↓	—	ND
Och6	115 ± 10%	—	—	—	—
Och7	108 ± 7%	—	—	—	—
Och8	98 ± 6%	—	—	—	—
Och9	112 ± 10%	—	—	—	—
Och10	119 ± 2%	—	—	—	—

^a^Average and standard error values of relative biofilm formation were calculated in at least three independent experiments.

^b^↓, >1.5-fold repressed, compared to pHMB1 vector; –, similar motility to pHMB1 vector; ↑, <1.5-fold changed, compared to pHMB1 vector.

^c^↓, unable to agglutinate yeast cells; –, able to agglutinate yeast cells.

^d^↓, formation of white-pink colony on Congo red indicator plates; –, similar color to pHMB1 vector; ↑, formation of dark red colony on Congo red indicator plates.

^e^ND; Not determined due to toxicity on growth.
