# Supplementary material for: Identification of novel sRNAs involved in biofilm formation, motility, and fimbriae formation in Escherichia coli
Source: Sci Rep. 2015 Oct 15;5:15287. doi: 10.1038/srep15287 (PMC4606813; doi:10.1038/srep15287)
Supplement: Supplementary Information [file srep15287-s1.pdf]

Supplementary Information

Identification of novel sRNAs involved in biofilm formation, motility, and fimbriae formation in *Escherichia coli*

Geunu Bak[1,3], Jungmin Lee[1,3], Shinae Suk[1], Daun Kim[1], Ji Young Lee[1], Kwang-sun Kim[2], Byong-Seok Choi[1], and Younghoon Lee[1,*]

[1]*Department of Chemistry, KAIST, Daejeon 305-701, Korea*

[2]*Superbacteria Research Center, KRIBB, Daejeon 305-806, Korea*

[3]These authors contributed equally to this work.

*Corresponding author.

Tel: 82-42-350-2832

Fax: 82-42-350-2810

E-mail: Younghoon.Lee@kaist.ac.kr

Supplementary Table 1. ***E. coli*** **sRNAs used for construction of sRNA-overexpressing plasmids**

	Name	Length (nt)	Characterized function	Hfq interaction	Biogenesis			Ref
					5'-end	3'-end	Rho-independent terminator	
1	ArcZ	120	Activation of RpoS, repression of *arcB*, *flhDC*	Yes	Yes	Yes	Yes	[1–5]
2	C0067	~125	Unknown	ND[c]	ND	ND	ND	[6]
3	C0293	~73	Unknown	ND	ND	ND	ND	[6]
4	C0299	~79	Unknown	ND	ND	ND	ND	[6]
5	C0343	~75	Unknown	ND	ND	ND	ND	[6]
6	C0362	~386	Unknown	ND	ND	ND	ND	[6]
7	C0465	~78	Unknown	ND	ND	ND	ND	[6]
8	C0614	~87	Unknown	ND	ND	ND	ND	[6]
9	C0664	~113	Unknown	ND	ND	ND	ND	[6]
10	C0719	~222	Unknown	ND	ND	ND	ND	[6]
11	CsrB	369	Antagonizing CsrA regulation	No	Yes	Yes	Yes	[1,2,7,8]
12	CsrC	245	Antagonizing CsrA regulation	No	Yes	Yes	Yes	[1,2,7,9]
13	CyaR	87	Induced by low glucose conditions, repression of *ompX, yqaE, nadE, luxS*	Yes	Yes	Yes	Yes	[2,10,11]
14	DicF	53	Inhibition of cell division, Interference with *ftsZ* translation	Yes	Yes	Yes	ND	[12–14]
15	DsrA	87	Activation of RpoS, repression of *hns*	Yes	Yes	Yes	Yes	[15–18]
16	EyeA (I001)	~75	Unknown	ND	ND	ND	ND	[19]
17	Ffs	114	4.5S RNA of Signal recognition particle (SRP)	No	Yes	Yes	ND	[20,21]
18	FnrS	119	Induced by unaerobic conditions, repression of *sodB, maeA, gpmA, folE, folX*	Yes	Yes	Yes	Yes	[22,23]
19	GadY	105	Antisense regulator of *gadXW*, repression of *gadW*, positive regulation of *gadX*	Yes	Yes	Yes	Yes	[24–26]
20	GcvB	205	Induced by high glycine, regulation of genes involved in amino acid metabolism (*oppA, dppA, gltI, livK, livJ, argT, cycA, sstT)*, repression of CsgD	Yes	Yes	Yes	Yes	[27–32]
21	GlmY	184	Decoy sRNAs for GlmZ	No	Yes	Yes	Yes	[7,33–36]
22	GlmZ	207	Increasing synthesis of *glmUS,* Stabilization of *glmS*	Yes	Yes	Yes	Yes	[1,2,34–37]
23	IS118	~190	Unknown	Yes	ND	ND	ND	[24,38,39]
24	IS128	~209	Unknown	ND	ND	ND	ND	[24]
25	McaS	94	Motility and biofilm regulator	Yes	Yes	Yes	Yes	[24,32,40]
26	IsrB	~160	Unknown, containing *azuC* ORF	No	ND	ND	ND	[24,41]
27	IsrC	~204	Unknown	ND	ND	ND	ND	[24]
28	IstR-1	75	*tisB* regulator	ND	Yes	Yes	Yes	[42,43]
29	IstR-2	140	SOS-regulated, mitigating IstR-1	ND	Yes	Yes	Yes	[42,43]
30	MgrR	99	Induced by low Mg^{2+}, PhoP, Regulator of LPS modification gene *eptB, ygdQ*	Yes	Yes	Yes	Yes	[38,44]
31	MicA	72	Repression of *ompA, ompX, phoPQ*	Yes	Yes	Yes	Yes	[1,10,45–47]
32	MicC	109	Repression of *ompC*	Yes	Yes	Yes	Yes	[24,48]
33	MicF	93	Repression of *ompF, lrp* antibiotics resistance	Yes	Yes	Yes	Yes	[49–52]
34	MicM	85	Repression of *chiP, dpiBA* by chbBCARFG decoy mRNA	Yes	Yes	Yes	Yes	[2,53–56]
35	OhsC	77	Regulator of *shoB* toxin	ND	Yes	Yes	ND	[2,57,58]

36	OmrA	88	Repression of outer membrane proteins (*cirA, fecA, fepA, ompT, gntP, ompR*), repression of *csgD, flhDC*	Yes	Yes	Yes	Yes	[1,2,59–62]
37	OmrB	82	Repression of outer membrane proteins (*cirA, fecA, fepA, ompT, gntP, ompR*), repression of *csgD, flhDC*	Yes	Yes	Yes	Yes	[1,2,59–62]
38	OxyS	110	Induced by oxidative stress, Repression of *fhlA, yobF-cspC, ybaYwrbA, rpoS*	Yes	Yes	Yes	Yes	[63,64]
39	PsrD	149~168	Unknown	Yes?[a]	Yes	Yes	Yes	[1]
40	PsrO	146~174	Unknown	ND	Yes	Yes	ND	[1]
41	RdlA	67	Antisense regulator of *ldr* toxin	ND	Yes	Yes	ND	[57]
42	RdlB	66	Antisense regulator of *ldr* toxin	ND	Yes	Yes	ND	[57]
43	RdlC	68	Antisense regulator of *ldr* toxin	ND	Yes	Yes	ND	[57]
44	RdlD	66	Antisense regulator of *ldr* toxin	ND	Yes	Yes	ND	[57,65]
45	RnpB	377	RNase P RNA	No	Yes	Yes	Yes	[66–68]
46	RprA	105	Activation of RpoS, repression of *csgD, ydaM*	Yes	Yes	Yes	Yes	[32,69–71]
47	RseX	91	Repression of *ompC, ompA*	Yes	Yes	Yes	Yes	[24,72]
48	RttR	171	Processed from *tyrT* transcript	ND	Yes	Yes	ND	[73]
49	RybA	202	*mntS* mRNA, Regulator of aromatic amino acid synthesis under oxidative stress	No	Yes	Yes	ND	[2,74,75]
50	RybB	81	Repression of *ompC, ompW*	Yes	Yes	Yes	ND	[2,76,77]
51	RybD	~103	Unknown	Yes	ND	ND	Yes	[38]
52	RydB	~68	Unknown	No	ND	ND	ND	[2,7]
53	RydC	64	Repression of ABC permease *yejABEF*	Yes	Yes	Yes	ND	[38,78]
54	RyeA	~249	Unknown	No	Yes	Yes	ND	[1,2]
55	SdsR	104	RpoS-dependent sRNA	Yes	Yes	Yes	Yes	[2,79]
56	RyeF	~388	Unknown	Yes	ND	ND	ND	[38]
57	RyfA	~304	Unknown	ND	ND	ND	ND	[2,7]
58	RyfB	280	Unknown, *shoB* mRNA	ND	Yes	Yes	ND	[57]
59	RyfD	143	Unknown	ND	Yes	Yes	ND	[57]
60	RyhB	90	Induced by iron limitation, repression of iron-containing proteins (*sdhCDAB, acnA, fumA, bfr, sodB*), repression of *cysE, shiA*	Yes	Yes	Yes	Yes	[1,2,80–83]
61	RyjA	140	Unknown	No	Yes	Yes	Yes	[1,2]
62	RyjB	90	Unknown	Yes	Yes	Yes	ND	[38,57]
63	SgrS	227	Repression of *ptsG, manXYZ,* containing *sgrT* ORF	Yes	Yes	Yes	Yes	[84–86]
64	SibA	144	Antisense regulator of toxin *ibs*	ND	Yes	Yes	ND	[2,58]
65	SibB	130	Antisense regulator of toxin *ibs*	ND	Yes	Yes	ND	[2,58]
66	SibC	141	Antisense regulator of toxin *ibs*	ND	Yes	Yes	ND	[2,58]
67	SibD	145	Antisense regulator of toxin *ibs*	ND	Yes	Yes	ND	[2,58]
68	SibE	144	Antisense regulator of toxin *ibs*	ND	Yes	Yes	ND	[58]
69	SokA	30	Pseudogene, antisense sRNA blocking mok/hok translation; the last 30/52 nt remain	ND	Yes	Yes	ND	[87]
70	SokB	56	antisense sRNA blocking mok/hok translation	ND	Yes	Yes	ND	[57,87]
71	SokC	55	antisense sRNA blocking mok/hok translation	ND	Yes	Yes	ND	[57,87]
72	SokE	59	antisense sRNA blocking mok/hok translation, missed start codon of mokE	ND	Yes	Yes	ND	[57,87]

73	SokX	56	Unknown, No hok toxic peptide	ND	Yes	Yes	ND	[57,87]
74	Spf	109	Repression of *galK*, broad role in catabolite repression	Yes	Yes	Yes	Yes	[88–90]
75	SraA	**~120**	Unknown	ND	ND	ND	ND	[1]
76	SroA	93	Unknown, leader region of *thiBPQ*	ND	Yes	Yes	ND	[91]
77	SroC	163	Unknown, leader region of *gltJKL*	Yes?[b]	Yes	Yes	Yes	[91]
78	SroD	86	Unknown, processed from *fadD*	ND	Yes	Yes	Yes	[91]
79	SroE	92	Unknown, processed from *gcpE*	ND	Yes	Yes	ND	[91]
80	SroG	149	Unknown, leader region of *ribB*	Yes?[a]	Yes	Yes	ND	[91]
81	SroH	161	Unknown, mutant is sensitive to envelope stress	ND	Yes	Yes	ND	[91,92]
82	SsrA	363	tmRNA	No	Yes	Yes	ND	[93]
83	SsrS	183	6S RNA, inhibition of σ^{70} RNA polymerase	No	Yes	Yes	ND	[94]
84	SymR	77	Antisense regulator of *symE*	ND	Yes	Yes	ND	[57,95]
85	T44	~136	Unknown, leader region of *rpsB*	ND	Yes	ND	ND	[7]
86	Tp2	~120	Unknown	ND	ND	ND	ND	[7]
87	Tpke11	~88	Unknown	ND	ND	ND	ND	[7]
88	Tpke70	~435	Unknown	ND	ND	ND	ND	[7]
89	pRNA	20	Unknown, synthesis from 6S RNA	ND	Yes	Yes	ND	[96]
90	Och1	255	Unknown	Yes	Yes	Yes	ND	[97]
91	Och2	188	Unknown	ND	Yes	Yes	Yes	[97]
92	Och3	192	Unknown	ND	Yes	Yes	Yes	[97]
93	Och4	241	Unknown	ND	Yes	Yes	ND	[97]
94	Och5	158	Unknown	ND	Yes	Yes	Yes	[97]
95	Och6	106	Unknown	Yes	Yes	Yes	ND	[97]
96	Och7	158	Unknown	Yes	Yes	Yes	ND	[97]
97	Och8	144	Unknown	ND	Yes	Yes	Yes	[97]
98	Och9	72	Unknown	Yes	Yes	Yes	ND	[97]
99	Och10	106	Unknown	Yes	Yes	Yes	ND	[97]

[a,b]Hfq interaction cited from other studies on *Klebsiella pneumoni*[a,98] and *Salmonella enterica serovar Typhimurium*[b,99].

[c]ND; Not determined.

Supplementary Table 2. Full sequences of cloned regions of each sRNA

sRNA	Vector	Preparation	Enzyme sites	Cloned sequence
ArcZ	pHM-tac	PCR	*Eco*RI/*Xba*I	GTGCGGCCTGAAAAACAGTGCTGTGCCCTTGTAACTCATCATAATAATTTACGGCGCAGCCAAGATTTCCCTGGTGTTGGCGCAGTATTCGCGCACCCCGGTCTAGCCGGGGTCATTTTTTagtggcttttgccacccacgctttc
C0067	pHMB1	PCR	*Eco*RI/*Xba*I	ataagagttagctgtattgttATGTCTGTGGCGAAATTGACTACCTTCGTTTTTTTGATTAAGAATGATTTTATTATCGTAAGTAAAATTACATGAATATTTAAAAAGGAAAACGACATGAAACCGAAGCACAGAATCAACATTCTccaatcataaaatatttccgtggagcattttattattgaatatagaggtttaactc
C0293	pHMB1	Gene synthesis	*Eco*RI/*Xba*I	agccggttttgtatcaactacTCACCCGGGACTCGCCAGGGGACAGCCAACAGGCATTGGGTGCAATCACCTTAGCGTTCAGGTACATGCGGAAtgtaaaaaaggccgcgagcgcggccccttcacatacatctttagtactgaga
C0299	pHMB1	Gene synthesis	*Eco*RI/*Xba*I	atgagcgtcgtcgccagaagGCCACGTGAGCACAAGATAAGAGAACGAAAAATCAGCAGCCTATGCAGCGACAAATATTGATAGCCTGAATCAGTATTGatctgctggcaagaacagactactgtatataaaaacagta
C0343	pHMB1	Gene synthesis	*Eco*RI/*Xba*I	atgaaccgattcctggcgatcCTGTTCCAGTGCCCGATCCGATCCCTCGCCCGCAACCCATGCCTGACCCACCACCCGATGAAGAACCGATTAAATtgtcgcatcgtgagcgtagatctgcgaggatacgcgcctg
C0362	pHMB1	PCR	*Eco*RI/*Xba*I	aagggatataatctcttttgAACTTTAAGCTGAAAATGGCGCTGTAAAAGGCGCCATTTTCATATTGTAGACAACGTAGGCTTTGTTCATGCCGGATGCGGCGTGAACGCCTTATCCGGCATGAAAACCCTTCAAATCCAATAGATTGCAGTGAACGTGTAGGCCTGATAAGCGTAGCGCATCAGGCAATGTTGCGTTTGTCATCAGTTTCAAATGGCGCTGTAAAAGGCGTCATTTTCATATTGTAGACAACGTAGGCTTTGTTCATGCCGGATGCGGCGTGAACGCCTTATCCGGCATGAAAACCCTTCAAATCCAATAGATTGCAGTGAACGTGTAGGCCTGATAAGCGTAGCGCATCAGGCAATGTTGCGTTTGTCATCAGTTCTAAATGGCGCTTTATAAAgtgccatttttttattgcgtaaccagacggcgtaatcgcgacac
C0465	pHMB1	Gene synthesis	*Eco*RI/*Xba*I	actcgttatcggcaaggagggGGGAAACTTTATTGCTGATGCCACCCGCCGCGAAATTGAAATAAAAAACCCGATGCGCAGATCATCGGGTTCATTTCAattgaggaaatcgggagaattacgccacttctgacgccgc
C0614	pHMB1	Gene synthesis	*Eco*RI/*Xba*I	attaccgaacacggcggaaagGGCCGGTGTCGATAATACGCTAAATAAACAATAATTACTCTCTTTTGCTTGACAAAAAGAGAGTTACTGGTGAGTATTGTTTTGCTGtgaagcacctgcgttgctcatgtaacacaagtcaacctgct
C0664	pHMB1	PCR	*Eco*RI/*Xba*I	atgccgatgtaggtgggctacTGTGCCTAAAATGTCGGATGCGACGCTGGCGCGTCTTATCCGACCTACGGGGACGCATGTGTAGGCCGGATAAGGCGTTTACGCCGCATCCGGCAATGGTGTCCAAATGCAACacgttttatccgttctggacttcacccgctaaccaacgcg
C0719	pHMB1	PCR	*Eco*RI/*Xba*I	aagaggactgaactgtaaaaTATAGGCGTTATACTTTACAGCAACAGTACGCCGCTAACGCAATTGCTACCTCTGGCATAACAAGTATATCGGGTAAGGGTTTCTGTTCCGCACACGCAGACGCAGAGTATCGTTAAGATGTCCATATTGTTGTTTTAGGCCCGCTAGTAATGCGCTACGGGTATTTAATATTGTTAAACCCTGATAATCGCTCCGGTTATTTCCGGGATAAATGTACTACCgcagttactatcatagccccgacaataaaacttgccgggg
CsrB	pHMB1	PCR	*Eco*RI/*Xba*I	aGTCGACAGGGAGTCAGACAACGAAGTGAACATCAGGATGATGACACTTCTGCAGGACACACCAGGATGGTGTTTCAGGGAAAGGCTTCTGGATGAAGCGAAGAGGATGACGCAGGACGCGTTAAAGGACACCTCCAGGATGGAGAATGAGAACCGGTCAGGATGATTCGGTGGGTCAGGAAGGCCAGGGACACTTCAGGATGAAGTATCACATCGGGGTGGTGTGAGCAGGAAGCAATAGTTCAGGATGAACGATTGGCCGCAAGGCCAGAGGAAAAGTTGTCAAGGATGAGCAGGGAGCAACAAAAGTAGCTGGAATGCTGCGAAACGAACCGGGAGCGCTGTGAATACAGTGCTCCCTTTTTTTATTcctgctatccttcgcggcagttttt
CsrC	pHMB1	PCR	*Eco*RI/*Xba*I	ATAGAGCGAGGACGCTAACAGGAACAATGACTCAGGATGAGGGTCAGGAGCGCCAGGAGGCGAAGACAGAGGATTGTCAGGAAGACAAACGTCCGGAGACGTAATTAAACGGAAATGGAATCAACACGGATTGTTCCGGCTAAAGGAAAAACAGGGTGTGTTGGCGGCCTGCAAGGATTGTAAGACCCGTTAAGGGTTATGAGTCAGGAAAAAAGGCGACAGAGTAATCTGTCGCCTTTTTTCTTtgcttgctttctgttagattccgcc
CyaR	pHM-tac	PCR	*Eco*RI/*Xba*I	GCTGAAAAACATAACCCATAAAATGCTAGCTGTACCAGGAACCACCTCCTTAGCCTGTGTAATCTCCCTTACACGGGCTTATTTTTTcgcgtaatacaatgaaataaaaggatttatttctggtcacgtccacacattgaccacatcgacaaaaaagcccctcgactgaggggcttcctgtttcta
DicF	pHMB1	PCR	*Eco*RI/*Xba*I	aTTTCTGGTGACGTTTGGCGGTATCAGTTTTACTCCGTGACTGCTCTGCCGCCCtttttaaagtgaattttgtgatgtggtgaatgcggctgagcgc
DsrA	pHMB1	PCR	*Eco*RI/*Xba*I	AACACATCAGATTTCCTGGTGTAACGAATTTTTTAAGTGCTTCTTGCTTAAGCAAGTTTCATCCCGACCCCCTCAGGGTCGGGATTTttttattgtgcattcaacgattcacttca
EyeA	pHMB1	Gene synthesis	*Eco*RI/*Xba*I	agaaacaccggggcagcagcgTCTATCACGCTTGCGTTGCTGACTTCTGCCAACTTGCGGCAAGCAAGGATAAAGAGTGCGACGGGCAGCCTCCTCagtatgcctgagtccaggcaggtaaaccggggaaggtcag
Ffs	pHMB1	Gene synthesis	*Eco*RI/*Xba*I	aGGGGGCTCTGTTGGTTCTCCCGCAACGCTACTCTGTTTACCAGGTCAGGTCCGGAAGGAAGCAGCCAAGGCAGATGACGCGTGTGCCGGGATGTAGCTGGCAGGGCCCCCACCCatttctgcctcccaccgtttcgtca
FnrS	pHMB1	PCR	*Eco*RI/*Xba*I	aGCAGGTGAATGCAACGTCAAGCGATGGGCGTTGCGCTCCATATTGTCTTACTTCCTTTTTTGAATTACTGCATAGCACAATTGATTCGTACGACGCCGACTTTGATGAGTCGGCTTTTTTTTgcctgttatttatcagcgtctaccc
GadY	pHMB1	PCR	*Eco*RI/*Xba*I	ACTGAGAGCACAAAGTTTCCCGTGCCAACAGGGAGTGTTATAACGGTTTATTAGTCTGGAGACGGCAGACTATCCTCTTCCCGGTCCCCTATGCCGGGTTTTTTTtatgtctgagtaaaactctataatc
GcvB	pHMB1	PCR	*Eco*RI/*Xba*I	ACTTCCTGAGCCGGAACGAAAAGTTTTATCGGAATGCGTGTTCTGGTGAACTTTTGGCTTACGGTTGTGATGTTGTGTTGTTGTGTTTGCAATTGGTCTGCGATTCAGACCATGGTAGCAAAGCTACCTTTTTTCACTTCCTGTACATTTACCCTGTCTGTCCATAGTGATTAATGTAGCACCGCCTAATTGCGGTGCTTTTTT

				TTaccttgcgatcgcgaattactgatc
GlmY	pHM-tac	PCR	*Eco*RI/*Xba*I	AGTGGCTCATTCACCGACTTATGTCAGCCCCTTCGGGACGTGCTACATAAAATACGAATGACGCACAACAAGGTGCCTGCCGTCCAACTTCTGATATCAGCGTAGCTATATCAACCATCGGGCGAAACGTCGAGTTAGGCACCGCCTTATTCCATAACAAAGCCGGGTAATTCCCGGCTTTGTTtatctgaacttcccctcggttagcatcaggctattcgcgtctgacgagagtaacaccttgaaacgctggcccgtttttccccgctcattacgacaactg
GlmZ	pHM-tac	PCR	*Eco*RI/*Xba*I	GTAGATGCTCATTCCATCTCTTATGTTCGCCTTAGTGCCTCATAAACTCCGGAATGACGCAGAGCCGTTTACGGTGCTTATCGTCCACTGACAGATGTCGCTTATGCCTCATCAGACACCATGGACACAACGTTGAGTGAAGCACCCACTTGTTGTCATACAGACCTGTTTTAACGCCTGCTCCGTAATAAGAGCAGGCGTTTTTTTatgtatcaggaaggccccggaggtgcttgcctccgggtgagaaggaactactgtggcgggttatt
IS118	pHMB1	PCR	*Eco*RI/*Xba*I	aGGTTCTGGAGGGGGTTTGTTGTGGGCAATGATGCATTTAAGTTATCGTCTGCAGATAGAGGAGATATTACAATAAACAACGAATCAGGGCATTTGATAGTCAATACCGCAATTCTATCAGGAGATATAGTCACTCTAAGAGGAGGAGAAATTAGGTTGGTATTATAGCTTGTGCGCGCCATGATTGGCGCGCAATTTaaacttagtgctttacatcgctattgtcttgatttctttgaattattttataaattaa
IS128	pHMB2	PCR	*Sma*I/*Xba*I	aagagagtaattattgtttaTTTAGCGTATTATCGACACCGGCCCTTTCCGCCGTGTTCGGTAATAAAATAACCTGGCTTATTAGTCCGAATTCAGACAAATATAAATAAATCCTGCTCAAAATTAAAAATTCTAACCGGTAAAAGATATTACTTAAACATGTAAATTCACTTTCCTTTAAAAAACAAAAAACCGCCAAAATCAGGCGGTTTTTTGTTGCTGGTCCGGTtcgcggcctttccagcaggttgtattaccgtagtaatgca
McaS	pHMB1	PCR	*Eco*RI/*Xba*I	acgatattaataatgtaacttatattttcgtgaaatctgtcactgaagaaaattggcaactaaaggttaaaaccgttataacacagtcACCGGCGCAGAGGAGACAATGCCGGATTTAAGACGCGGATGCACTGCTGTGTGTACTGTAGAGTCTGGCGGATGTCGACAGACTCTATTTTTTTATgcagttttaactttgcagatagccgcatt
IsrB	pHMB1	PCR	*Eco*RI/*Xba*I	atgtcatattataagcgcaaaGACAATAACACCTGTATAACAAATGGTCGGAGTGCCGCGATGAAACTGCGCAAAATCCTGAAAAGTATGTTCAATAACTATTGCAAGACGTTCAAAGACGTACCGCCAGGCAATATGTTCCGATAACAAAAAACCTGCTCCGGCAGGTTTTTTTGTGTCCtgatgacggtggatgtgactatcgctgaagatgtgaagtc
IsrC	pHMB1	PCR	*Eco*RI/*Xba*I	atagctcaataatagaataaaACGATCAATATCTATTTTATCGATCGTTTATATCGATCGATAAGCTAATAATAACCTTTGTCAGTAACATGCACAGATACGTACAGAAAGACATTCAGGGAACAACAGAACCACAATTCAGAAACTCCCACAGCCGGACCTCCGGCACTGTAACCCTTTACCTGCCGGTATCCACGTTTGTGGGTACCGGCTTTTTTATTCACCctcaatctaaggaaaagctgatgaaa
IstR-1	pHMB1	Gene synthesis	*Eco*RI/*Xba*I	aGTTGACATAATACAGTGTGCTTTGCGGTTACCAGCCGCAGGCGACTGACGAAACCTCGCTCCGGCGGGGTTTTTTgttatctgcaattcagtacaaaacg
IstR-2	pHMB1	PCR	*Eco*RI/*Xba*I	aGCACTAAATACGTCAAAATTCGTGCCGAAATTGCGCGTTCTGCGCGGAACACGTATACTTTCAGTGTTGACATAATACAGTGTGCTTTGCGGTTACCAGCCGCAGGCGACTGACGAAACCTCGCTCCGGCGGGGTTTTTTgttatctgcaattcagtacaaaacg
MgrR	pHMB1	PCR	*Eco*RI/*Xba*I	aGATTCGTTATCAGTGCAGGAAAATGCCTGTTAGCGTAAAAGCAAAACACAAATCTATCCATGCAAGCATTCACCGCCGGTTTACTGGCGGTTTTTTTTcgccgtcataaaaatcaggcccctt
MicA	pHMB2	PCR	*Sma*I/*Xba*I	aGAAAGACGCGCATTTGTTATCATCATCCCTGAATTCAGAGATGAAATTTTGGCCACTCACGAGTGGCCTTTTTCTTTTctgtcaggcgtgtttttccagccac
MicC	pHMB1	PCR	*Eco*RI/*Xba*I	aGTTATATGCCTTTATTGTCACAGATTTTATTTTCTGTTGGGCCATTGCATTGCCACTGATTTTCCAACATATAAAAAGACAAGCCCGAACAGTCGTCCGGGCTTTTTTTttagaattggataatccttatccagag
MicF	pHMB1	PCR	*Eco*RI/*Xba*I	aGCTATCATCATTAACTTTATTTATTACCGTCATTCATTTCTGAATGTCTGTTTACCCCTATTTCAACCGGATGCCTCGCATTCGGTTTTTTTTaccttctttacacactttcatta
MicM	pHMB1	PCR	*Eco*RI/*Xba*I	ACACCGTCGCTTAAAGTGACGGCATAATAATAAAAAAATGAAATTCCTCTTTGACGGGCCAATAGCGATATTGGCCATTTTTTTagcgcaacatttgcggcaaattccc
OhsC	pHMB1	Gene synthesis	*Eco*RI/*Xba*I	actgatccataatcgctgttGTTGAGGGTGCATGCTGCACAAAATTAAAGTTAAAAAGTAAAACCCCCGTTCCTTACCAGTTCGGGGGTTTTACTTTttaaagagaacggtattatttttaa
OmrA	pHMB1	PCR	*Eco*RI/*Xba*I	aCCCAGAGGTATTGATTGGTGAGATTATTCGGTACGCTCTTCGTACCCTGTCTCTTGCACCAACCTGCGCGGATGCGCAGGTTTTTTTTcgcacctaatttactgtcgctcgcg
OmrB	pHMB1	PCR	*Eco*RI/*Xba*I	aCCCAGAGGTATTGATAGGTGAAGTCAACTTCGGGTTGAGCACATGAATTACACCAGCCTGCGCAGATGCGCAGGTTTTTTTTgccggtcatcaatctgtaacagtaa
OxyS	pHMB1	PCR	*Eco*RI/*Xba*I	aGAAACGGAGCGGCACCTCTTTTAACCCTTGAAGTCACTGCCCGTTTCGAGAGTTTCTCAACTCGAATAACTAAAGCCAACGTGAACTTTTGCGGATCTCCAGGATCCGCTtttttttgccataaaaaagcccggcgataagc
PsrD	pHMB1	PCR	*Eco*RI/*Xba*I	aTAGGCATATTTTTTTCCATCAGATATAGCGTATTGATGATAGCCATTTTAAACTATGCGCTTCGTTTTGCAGGTTGATGTTTGTTATCAGCACTGAACGAAAATAAAGCAGTAACCCGCAATGTGTGCGAATTATTGGCAAAAGGCAACCACAGGCTGCCTTTTTCTTTgactctatgacgttacaaagttaat
PsrO	pHM-tac	PCR	*Eco*RI/*Xba*I	ATCTTCTGCGCATCCTCGCGACTAATGACAACCCTAACCCAGCTCTATGTGGGTAAAGCCTCTCATTAGCCGCGCGAACCTCTGCAACGGAAGATCATTCATAGCAACAATACATTAGTTTCCAGTGAATTGCTGCCGTCAGCTTGAAAAAAGGGGCCACTCAGGCCCCCTTTTctgaaactcgcaagaattagcgacgcagacccaggcgctcgatgagctgggtgtaacgtgctacgtctttacgtttcaggtagtcgagcagtttacgacg
RdlA	pHMB1	Gene synthesis	*Eco*RI/*Xba*I	atttgcggctattcttgaattGTTCTGGTTCAAGATTAGCCCCCGTTCTGTTGTCAGGTTGTACCTCTCAACGTGCGGGGGTTTTCTCtttccagcaaccaatgccaccagggataaagcccccgcaa
RdlB	pHMB1	Gene synthesis	*Eco*RI/*Xba*I	aGTCTGGTTTCAAGATTAGCCCCCGTTCTGTTGTCAGGTTTTACCTCTCAACGTGCGGGGGTTTTCTctttccagcaaccaatgccaccagggataaagcccccgca
RdlC	pHMB1	Gene synthesis	*Eco*RI/*Xba*I	aGTCTGGTTTCAAGATTAGCCCCCGTTTTGTTGTCAGGTTTTACCTCTCAACGTGCGGGGGTTTTCTCTttccagcaaccaatgccaccagggataaagccccgcaaca
RdlD	pHMB1	Gene synthesis	*Eco*RI/*Hin*dIII	aGTCTAGAGTCAAGATTAGCCCCCGTGGTGTTGTCAGGTGCATACCTGCAACGTGCGGGGGTTTTctctctccagcaaccaatgccaccagggataaagcccccgca

RnpB	pHMB1	PCR	*Eco*RI/*Xba*I	GAAGCTGACCAGACAGTCGCCGCTTCGTCGTCGTCCTCTTCGGGGGAGACGGGCGGAGGGGAGGAAAGTCCGGGCTCCATAGGGCAGGGTGCCAGGTAACGCCTGGGGGGGAAACCCACGACCAGTGCAACAGAGAGCAAACCGCCGATGGCCCGCGCAAGCGGGATCAGGTAAGGGTGAAAGGGTGCGGTAAGAGCGCACCGCGCGGCTGGTAACAGTCCGTGGCACGGTAAACTCCACCCGGAGCAAGGCCAAATAGGGGTTCATAAGGTACGGCCCGTACTGAACCCGGGTAGGCTGCTTGAGCCAGTGAGCGATTGCTGGCCTAGATGAATGACTGTCCACGACAGAACCCGGCTTATCGGTCAGTTTCACCTgatttacgtaaaaacccgcttcggcgggtttttgcttttg
RprA	pHMB1	PCR	*Eco*RI/*Xba*I	ACGGTTATAAATCAACATATTGATTTATAAGCATGGAAATCCCCTGAGTGAAACAACGAATTGCTGTGTGTAGTCTTTGCCCATCTCCCACGATGGGCTTTTTTTTaacatttttccgcttcgctacctcg
RseX	pHMB1	PCR	*Eco*RI/*Xba*I	aTTTTTATTATTCTGTGTCATGATGCTTCCGTTATTAGCCTTTTATCGTCTTGTTTATATTTTTTGGGCCGGCATGATGCCGGCTTTTTTTTatgccttcattaatgtgcgcctgat
RttR	pHMB1	PCR	*Eco*RI/*Xba*I	atcccccaccaccatcactttCAAAAGTCCCTGAACTTCCCAACGAATCCGCAATTAAATATTCTGCCCATGCGGGGAAGGATGAGAAGCTTCGACCAAGGTTCGACTCGAGCGCCAGCGAGAGAGCGTTGCCGCAGGCAACGACCCGAAGGGCGAAGCGCGCAGCGCTGAGTAATCCTTCCCCCACCACCAtcactttcaaaagtccctgaactctcaagcgaatccgcaa
RybA	pHMB1	PCR	*Eco*RI/*Xba*I	agcctgtgctatatctgtatgtaatgcaaTCATCCCTCAAGGATCGACGGGATTAGCAAGTCAGGAGGTCTTATGAATGAGTTCAAGAGGTGTATGCGCGTGTTTAGTCATTCTCCCTTTAAAGTACGGTTAATGCTGCTCTCTATGTTGTGCGATATGGTCAACAACAAACCGCAGCAAGATAAACCTTCCGATAAATAGCGGCGTCGCGGTACGCCGCTTCACtcctgctttcatgcaggcataacgcgttttggtctgaaaa
RybB	pHMB1	PCR	*Eco*RI/*Xba*I	ACTGCTTTTCTTTGATGTCCCCATTTTGTGGAGCCCATCAACCCCGCCATTTCGGTTCAAGGTTGATGGGTTTTTTGTTatctaaaacttatctactaccctgc
RybD	pHMB1	PCR	*Eco*RI/*Xba*I	ATATCTGTAATAAGAAATAGCCCTCGCCGCTTCCCTCTACAGGAATGGCGAAGGGCTGTCGGTTTCGACATGGTTGGCCATCGTATGATGGCCTTTTTTgtgcttatcgcgatgattttcgctg
RydB	pHMB1	PCR	*Eco*RI/*Xba*I	actaatcgcagtttgtgttaaaacggcgggttagctttatgagATTATTCTTATCGCCCCTTCAAGAGCTAAGCCACTGAGAGTGCCGGAGATAAGCGCCGGATGGGGTAGAAACCCTTAAGCctgtgtcgcacagacttaagggttttcttatttct
RydC	pHMB1	PCR	*Eco*RI/*Xba*I	aCTTCCGATGTAGACCCGTATTCTTCGCCTGTACCACGGGTCGGTTTTAGTACAGGCGTTTTCTTtaaatatccttcaggaacgtttacg
RyeA	pHM-tac	PCR	*Eco*RI/*Xba*I	AAAGTCAGCGAAGGAAATGCTTCTGGCTTTTAACAGATAAAAAGAGACCGAACACGATTCCTGTATTCGGTCCAGGGAAATGGCTCTTGGGAGAGAGCCGTGCGCTAAAAGTTGGCATTAATGCAGGCTTAGTTGCCTTGCCCTTTAAGAATAGATGACGACGCCAGGTTTTCCAGTTTGCGTGCAAAATGGTCAATAAAAAGCGTGGTGGTCATCAGCTGAAATGTTAAAAACCGCCCGTTCTGGTGAaagaactgaggcggttttttttattggaaatcaaaaggctattttaggtaattaacagagtttttcagctcgttctataaacggtgccagactcatttttcgccgggattgttaggatcatcaatctgaatcaccgaaatgggttgggcattggtcttcccactggcaacttccttttgtgcgatatcgtttaaaggatactgcacgagggtactcggattaataacatacaaagcattacccggtcggcaagtcagcatcacctcttcgc
SdsR	pHM-tac	PCR	*Eco*RI/*Xba*I	GGCAAGGCAACTAAGCCTGCATTAATGCCAACTTTTAGCGCACGGCTCTCTCCCAAGAGCCATTTCCCTGGACCGAATACAGGAATCGTGTTCGGTCTCTTTTTatctgttaaaagccagaagcatttccttcgctgactttat
RyeF	pHMB1	PCR	*Eco*RI/*Xba*I	ATTTTTACCGTTGCATCATGTCGCCCAATATGATGCTTGCTCGTACCAGGCCCCTGCAATTTCAACAGGGGCCTTTTTTTATCCCTGAACAGTATAAAAAACGAACGATAACCGTGATCTGTTGAGCGGGTGACAGTGCGCATAGCGTTGTGCTAAAAATATTGTATATATTCACATTAATTATGGGATTTAAATTACTAAAACTGATAAATATATATTCTAAATAGCAACTGGGTTATTCCTTAGCAATTAATGATTACATTGTAATAAATCATATTCTTTATCGATTGTTTCAGGCAGTGTTGTGCCTAATTATGCAAGCGGTTAATTCGTTGTATATTTAATTATACAATGATTTCGGTGTCCAGTAATTTAATTAGAGGAATCT
RyfA	pHMB1	PCR	*Eco*RI/*Xba*I	aGCGGCCCTTTCCGCCGTCTCGCAAACGGGCGCTGGCTTTAGGAAAGGATGTTCCGTGGCCGTAAATGCAGGTGTTTCACAGCGCTTGCTATCGCGGCAATATCGCCAGTGGTGCTGTCGTGATGCGGTCTTCGCATGGACCGCACAATGAAGATACGGTGCTTTTGTATCGTACTTATTGTTTCTGGTGCGCTGTTAACCGAGGTAAATAATAACCGGAGTCTCTCCGGCGACAATTTACTGGTGGTTAACAACCTTCAGAGCAGCAAGTAAGCCCGAATGCCGCCCTTTGGGCGGCATATTTTagattatccgattctgtttaaagtc
RyfB	pHMB1	PCR	*Eco*RI/*Xba*I	aCGTTATTGAAGATTTTGCTGTGCTTTACACCATGCCACAGAATTCCCCCATTGAAACGAGTGGTGTCGTCAAAGCTCTGGTGTGGAGTGCAGCATGCACCCTCAATAACTCGCACGTTCAGTTTTGGGGAGATGTAAGGGCTAATCTGAATGGCTGCATTCCTTGTTTAAGGAAAAACGAATGACTGATTGCCGATACCTGATTAAACGGGTCATCAAAATCATCATTGCTGTTTTACAGCTGATCCTTCTGTTCTTATAACACAAGGAAACGTACTTAAGGTGCGTCCGGTGAACCAGTCGGACGCACCTTTAATAACtataaataagtgtctgggcagatactatataaattaactt
RyfD	pHMB1	PCR	*Eco*RI/*Xba*I	AATCAAGACGATCCGGTACGCGTGATTTTCTTTTCACATTAATCTGGTCAATAACCTTGAATAATTGAGGGATGACCTCATTTAATCTCCAGTAGCAACTTTGATCCGTTATGGGAGGAGTTATGCGTCTGGATCGTCTTACTaataaattccagcttgctcttgccgatgcccaatcactt
RyhB	pHM-tac	PCR	*Eco*RI/*Xba*I	GCGATCAGGAAGACCCTCGCGGAGAACCTGAAAGCACGACATTGCTCACATTGCTTCCAGTATTACTTAGCCAGCCGGGTGCTGGCTTTTtttttgatctttcgttctcaatttatccacgggagtgcttgtgttcgttatgcgcactccagtaggaaccacgtccgctttgcgctaaggtgtaaataac
RyjA	pHMB1	Gene synthesis	*Eco*RI/*Xba*I	ATCAACACCAACCGGAACCTCCACCACGTGCTCGAATGAGGTGTGTTGACGTCGGGGGAAACCCTCCTGTGTACCAGCGGGATAGAGAGAAAGACAAAGACCGGAAAACAAACTAAAGCGCCCTTGTGGCGCTTTAGTTT
RyjB	pHMB1	Gene synthesis	*Eco*RI/*Xba*I	aTCATCCGTCGTTGACTCCATGCCGATTCGGGTTAATCTGGTAGCGATCCCCGTCGATACTTTTGACGAAGGCGGCAGGGATCGCAGAAGGgttattgctctttgccggggataagcgctctgcttatccc
SgrS	pHMB1	PCR	*Eco*RI/*Xba*I	aGATGAAGCAAGGGGGTGCCCCATGCGTCAGTTTTATCAGCACTATTTTACCGCGACAGCGAAGTTGTGCTGGTTGCGTTGGTTAAGCGTCCCACAACGA

				TTAACCATGCTTGAAGGACTGATGCAGTGGGATGACCGCAATTCTGAAAGTTGACTTGCCTGCATCATGTGTGACTGAGTATTGGTGTAAAATCACCCGCCAGCAGATTATACCTGCTGGTTTTTTTTTattctcgccgcgctaaaaagggaac
SibA	pHMB1	Gene synthesis	*Eco*RI/*Xba*I	aGAGGGTTAGGGAGAGGTTTCCCCCTCCCCCTGGTGTTCTTAGTAAGCCTGGAAGCTAATCACTAAGAGTATCACCAGTATGATGACGTGCTTCATCATAACCCTTTCCTTATTAAAAGCCCTCTTCTCCGGGAGAGGCTTTCCCgtttcagcgtcccgctgaaatcatc
SibB	pHMB1	Gene synthesis	*Eco*RI/*Xba*I	aGAGGGTAGAGCGGGGTTTCCCCCGCCCTGGTAGTCTTAGTAAGCGGGGAAGCTTATGACTAAGAGCACCACGATGATGAGTAGCTTCATCATGACCCTTTCCTTATTTATGGCCCCTTCCTCGGGAGGGGctttcccgtttcagcgtcccgctgaaatcgt
SibC	pHMB1	Gene synthesis	*Eco*RI/*Xba*I	AAGGGTAAGGGAGGATTGCTCCTCCCCTGAGACTGACTGTTAATAAGCGCTGAAACTTATGAGTAACAGTACAATCAGTATGATGACAAGTCGCATCATAACCCTTCTCCTTCAAGCCCTCGCTTCGGTGAGGGCTTTACCgttacagccccatgctgccctgcca
SibD	pHMB1	Gene synthesis	*Eco*RI/*Xba*I	ACAAGGGTGAGGGAGGATTTCTCCCCCCTCTGATTGGCTGTTAATAAGCTGCGAAACTTACGAGTAACAACACAATCAGTATGATGACGAGCTTCATCATAACCCTTTCCTTCTGTAAGGCCCCCTTCTTCGGGAGGGGCTTTCCcgtttcagccccctgctgactcccc
SibE	pHMB1	Gene synthesis	*Eco*RI/*Xba*I	ACAAGGGTAAGGGAGGATTTCTCCCCCCTCTGATGAGTTGTTAGTAAGTCGGGAAACTTAACAGTAACAACACAACCAGTATGATGACGAGCTTCATCATAACCCTTTCCTTATACAAGGCCCCTTCTTCGGGAGGGGCTTTCCcgtttcagccccttgctgcccccccg
SokA	pHMB1	Gene synthesis	*Eco*RI/*Xba*I	AACTTTGATTTATAGTCAGGTGGGGCTTTTctctgtctgcctttcggtgaatacctgagacaaacagtct
SokB	pHMB1	Gene synthesis	*Eco*RI/*Xba*I	aGCTAGGTTCATTCGTTGGCCTCGGTTGATAGAAATATCGGTCGGGGCCTTCGTCTTtctgattcccggttagcctgaaaacagaaagtctcaggca
SokC	pHMB1	Gene synthesis	*Eco*RI/*Xba*I	aGTTCAGCATATAGGAGGCCTCGGGTTGATGGTAAAATATCACTCGGGGCTtttctctatctgccgttcagctaatgcctgagacagacagcctca
SokE	pHMB1	Gene synthesis	*Eco*RI/*Xba*I	AAGATGAAGCCCCAGGAGATATTTCTATCAACCCTGGGGCTGCCACTCCAAACCCGAACaatttggatggtagtcccttcttcgcatggaggcaatata
SokX	pHMB1	Gene synthesis	*Eco*RI/*Xba*I	aTTTGGGTTCGAACGCTGGCCTCAGGTTGATAGAAATATCGCCTGGGGCTTTTGTCCatctggaacctcgcgaatgcttaacgccagacagcctcaa
Spf	pHMB1	PCR	*Eco*RI/*Xba*I	aGTAGGGTACAGAGGTAAGATGTTCTATCTTTCAGACCTTTTACTTCACGTAATCGGATTTGGCTGAATATTTTAGCCGCCCCAGTCAGTAATGACTGGGGCGTTTTTTATTgggcgaaagaaaagatccgtaatgcctgatgcgc
SraA	pHMB1	Gene synthesis	*Eco*RI/*Xba*I	atatggggatgtttcccccaCATTCAACGCCGAGAATAGAGGAAAAATTAAAGGGGAGATAAAATCCCCCCTTTTTGgttactaattgtatgggaatggtt
SroA	pHMB1	Gene synthesis	*Eco*RI/*Xba*I	aCACTAACGCCGTTCTCAACGGGGTGCCACGCGTACGCGTGCGCTGAGAAAATACCCGTCGAACCTGATCCGGATAACGCCGGCGAAGGGATTTGAGGCTCCTTctcaagtcctttgccactctttttt
SroC	pHMB1	PCR	*Eco*RI/*Xba*I	AAGGCACTGAACTAATTACAAGAACCAGGGGCGGAAATTCCAGCCCTCTCGATTGTTACGTAGCACGGACAGACTATACGCCTGATGGTCGTTCCCCATCGGGCCTGAAAACCGCAATACGCTGGGTAACAATCTTCGAGGGTAGCAGTTAACGCTGCTACCCTCTTTTTTCTggagtagatttatgtctatagactg
SroD	pHMB1	Gene synthesis	*Eco*RI/*Xba*I	agaaaaattttgcgacgagaaTTACGTGACGAAGCGCGCGGCAAAGTGGACAATAAAGCCTGAGCGTTAAGTCAGTCGTCAGACGCCGGTTAATCCGGCGTTTTTTTtgacgcccactaaagagaaaacaat
SroE	pHMB1	PCR	*Eco*RI/*Xba*I	agttcagcaggttgaaaaataATAACGTGATGGGAAGCGCCTCGCTTCCCGTGTATGATTGAACCCGCATGGCTCCCGAAACATTGAGGGAAGCGTTGAGGGTTCATTTTTATattcagaaagagaataaacgtggca
SroG	pHMB1	PCR	*Eco*RI/*Xba*I	aGCTTATTCTCAGGGCGGGGCGAAATTCCCCACCGGCGGTAAATCAACTCAGTTGAAAGCCCGCGAGCGCTTTGGGTGCGAACTCAAAGGACAGCAGATCCGGTGTAATTCCGGGGCCGACGGTTAGAGTCCGGATGGGAGAGAGTAACGattctgtcgggcatggacccgctcacgttattttggctat
SroH	pHMB1	PCR	*Eco*RI/*Xba*I	aGAAAATAAGAACACATGTTCTCATCTTCCAGGATGCAGCAGACTGAAGAAATTCAGACATCCCGCAACCTGCGATTATCGCAAGGTCAAGGCAAAGTCCGGTAATGGCGTTCTGAATACCAGAGATAATTCTCTGGCGAAACCCACCTTAAGGTGGGTTTTgttattttgagggctgaggaagctg
SsrA	pHMB1	PCR	*Eco*RI/*Xba*I	aGGGGCTGATTCTGGATTCGACGGGATTTGCGAAACCCAAGGTGCATGCCGAGGGGCGGTTGGCCTCGTAAAAAGCCGCAAAAAATAGTCGCAAACGACGAAAACTACGCTTTAGCAGCTTAATAACCTGCTTAGAGCCCTCTCTCCCTAGCCTCCGCTCTTAGGACGGGGATCAAGAGAGGTCAAACCCAAAAGAGATCGCGTGGAAGCCCTGCCTGGGGTTGAAGCGTTAAAACTTAATCAGGCTAGTTTGTTAGTGGCGTGTCCGTCCGCAGCTGGCAAGCGAATGTAAAGACTGACTAAGCATGTAGTACCGAGGATGTAGGAATTTCGGACGCGGGTTCAACTCCCGCCAGCTCCAccaaaattctccatcggtgattaccaga
SsrS	pHMB1	PCR	*Eco*RI/*Xba*I	agaagacaaaATTTCTCTGAGATGTTCGCAAGCGGGCCAGTCCCCTGAGCCGATATTTCATACCACAAGAATGTGGCGCTCCGCGGTTGGTGAGCATGCTCGGTCCGTCCGAGAAGCCTTAAAACTGCGACGACACATTCACCTTGAACCAAGGGTTCAAGGGTTACAGCCTGCGGCGGCATCTCGGAGATTCccttcttatctggcaccagccatga
SymR	pHMB1	Gene synthesis	*Eco*RI/*Xba*I	AGTCATAACTGCTATTCTCCAGGAATAGTGATTGTGATTAGCGATGCGGGTGTGTTGGCGCACATCCGCACCGCGCTaaatacctgtatatatcatcagtaa
T44	pHMB1	PCR	*Eco*RI/*Xba*I	atttgtggtataaagcgcgcCGGACTTCCGATCCATTTCGTATACACAGACTGGACGGAAGCGACAATCTCACTTTGTGTAACAACACACACGTATCGGCACATATTCCGGGGTGCCCTTTGGGGTCGGTAATATGGGATACGTGGAGGCATAACCccaacttttatatagaggttttaatcatggcaactgtttc
Tp2	pHMB1	PCR	*Eco*RI/*Xba*I	ataattttaccagaaaaatcACTAATTCTTTCGTTGCTCCAGACGACGCAGAGAACGCTCACGGCGGCTCTCTTCACGACTTCTGTCGAGCAAAATTTCTTCGATAAAGGCCAGATGGCGATGCGATGCTTCGCGCGCTTCTTCCGGCTTACCGGCCATAATCGCTTCAAATATGCGGGTgcggtgactactcaccagcggcagcatctcgcgacgcga
Tpke11	pHMB1	Gene synthesis	*Eco*RI/*Xba*I	aagtcaaagacaaaaaataaTCGCCCTATAAACGGGTAATTATACTGACACGGGCGAAGGGGAATTTCCTCTCCGCCCGTGCATTCATCTAGGGGCAATTTAAAAAAGAtggctaagcaagattattacgagattttaggcgtttccaaa
Tpke70	pHMB1	PCR	*Eco*RI/*Xba*I	aaggaaaaaaccttaagaaaAAAGCCATAAAAACCATGAGGTTATTATGGCCGATTTGAGGAGGGAAAGAGTAAGAGCAGTTTGTTAAATGTACAACGACGATTCT

				CCCACCGGGCGCGTTTTAAAGCGACGGTGGATCCAGAGGTACTGCTCCGGTGCGCGCATGATCTCTTTCTCGATAATCTTGTTCATATAGGCAGCGGCTTGATTTTCATCTGTCGGGTAGCCTTCCATCTCTGGGGTGATGAACAAACGATATCCGCTGTAATCCGCTTTTCTTACCATCGTTACGGTCAACATGGCTGCGCCAGAGAGACGGGAGAGAACATAGGTGCCATTGGTTGTGGCGACATTTTCCACCGCAAAGAACGGCGCGAAGGAGCTGCCTTTACGACCATAATCCTGATCGGGAGCAAACCATACCGCTTCACCTTTCTTCAGTGCACCGACAATGCCgcgcagattatttctgccgatcatcgctttgttagagcgc
pRNA	pHMB1	Oligo Annealing	*Eco*RI/*Xba*I	ATCGGCTCAGGGGACTGGCC
Och1	pHMB1	PCR	*Eco*RI/*Xba*I	aTGCCTTTGGTAGAGGAAAGTGCTAAATAATAATCAATTGTTAAATTATTGTGCATTTCACTACTGGAACTGTAATCAGAAAAGATAGACATGCTTAGCCAATCTCTATTTGATTGAATTGAAAGATGTTTGTTAAGGCATGGATGCAAGCTATAGATTCTGATACGGTCAATAAAAGAGAATTGCTTAACAATTTTGCAAAATGTATTGGCGAGTAAGAACCGCATTTGGTACTTTCCGGGCAACCGCCAGACGAttctttattggtaatgagaataattaacaattaaagagcg
Och2	pHMB1	PCR	*Eco*RI/*Xba*I	aTGCGCATCAGTAATAAATGACACACAGCAAAATGAATCCGTTTATTTGGGTACTTATAATCCTGATGACGCTAGACGCGCTGCGGGAATTGGCTGGCGCTTCGTCTATTTTAGGATGGCTATTAACGTTGGTTTGAGCTGGCAATAAGTCCGGACGGGTATTTACCGCAGTCCGGACTTATTTTTCAGgcgtgcagacgacgatgcaaacgcg
Och3	pHMB1	PCR	*Eco*RI/*Xba*I	ATATATAATTTATATTATTCAGGCAATGAATTACTTTTGCAAGCCATCGCATTCTCTTATGTTATTAATGAGTTATGCTGATTTGTTAAGCAGTTTTATCAGGCTTGAAATGGCGTCCAGCCCCGACAGGTGAATCGTCGGGGCTGATTTTTTCTTATTATTGCAGCAGAAACTTCTCGAGGAACTGGCGGGTGCGAGGCTGCTCGGGGTCGGCAAAT
Och4	pHMB1	PCR	*Eco*RI/*Xba*I	aCGATATATGTTTATTTTATGTAAAATCAATTTATGTAAAAAGTCACATCATTGTAGTTAAAAAGGTTGAGTTAGATCGCAGAAACGGGTACATATAGCCCCGCAAACGTGACCACGCCCGCAGATATTACTTAAATCAGAGCCATAGAGGCCACGCAGGCGAGGCATCAATCTTTACGATCTGTATAAAGACGGATTGTTGATGATGTGTTAAAATTGATGTAAACAAATTGTGAAgtgaatgtgcttccggggaaaataagtgacttcattacaa
Och5	pHMB1	PCR	*Eco*RI/*Xba*I	ATCCTTGTCCGCTTAACCGTTTCAGTCAGCCTATCCTTGATGAAACCGCGAGCAAAGATAGGTGATTACGTCATGGTTTTACAGAAAATTACAGAAAAAGGAGGCAATATCGGGTAAAGGCATTAGCCCGACGAATACGTCGGGCTACAAATATTATTgtgctgcaggtgttttagcgggttg
Och6	pHMB1	PCR	*Eco*RI/*Xba*I	aGCGCCTTGAAAAGAGGCCAAAGCCTGCCATTTTTAGCTCAATTCGGCGATGACCTGGATCAATCGTCCCGGCCTGCTTTTATCAACTGCATAATCAATCAAAATTAccgaaatttcatgcataatcacataaatcacttttgctta
Och7	pHMB1	PCR	*Eco*RI/*Xba*I	aGGTGAGCACTGAACAATTAAGAACAGCGTATTACATTTACTTTCCCCAAGATATGCGAAGAGCTTTCCTGAAACATTAACCGATCGTATAAAACGAATGCCTGATAGTACTCCTGTCCGGCATTGTTATCACAAGTAGACTACGCTTATCACAGCTTTcccatcaagttcgatttttctgaaccgcctaaatcattc
Och8	pHMB1	PCR	*Eco*RI/*Xba*I	aCACGATCACTCTAAGAGGACATTCGCCTTGGACACACCCAGTAGATACTGGCTCACTATCCTGTCATCCAGGATCAACTCCTAAGGCTATCCCTTTTTGCTGATAGCCTTAGCGGTTGTCAGCGACCTCAATTTTTCCCGTCGCgctgagtcaggctgtttaatggtct
Och9	pHMB1	PCR	*Eco*RI/*Xba*I	ACCATCGTGCAAAAGGGCTGCACCACGATGTGAATGTTGCACCAATATAGTGCTTCAATGGAAACATTAAGCAccatgttggtgcaatgacctttgga
Och10	pHMB1	PCR	*Eco*RI/*Xba*I	ATACTAGCCACTGAAAATGCCGGTTCACTTTCTTCGAATCGGCTTTCAATGTGTATTTCACACAAATTAATCAACTTCCCCTTCCGAGGATCTGGCCTGAAAGGTCggataagatatgttaaacagtattttagtcatactctgct

Predicted sRNA sequences were indicated by capital letters.
An extra adenine nucleotide to facilitate efficient transcription was indicated by underline.

Supplementary Table 3. Oligonucleotides sequences used in this study

Name	Sequence (5' to 3')	Used for
BamHIss6F	CGGGATCCATAAATGTGAGCGGATAACATTGACATTGTGAGCGG	Constructing pHMB1
HindIIIss6R	CCCAAGCTTGTCGACTCTAGAATTATATTGTTATCCGCTCACAATGTC	Constructing pHMB1
pHMB1oligo1	AATTCGAGCTCGCGTCTAGAGCCGGCAAGCTTGGAGTACGTAAAAACCCGCTTCGGCGGGTTTTTGCTTTTGGAGGGGC	Constructing pHMB1
pHMB1oligo2	AGCTGCCCCTCCAAAAGCAAAAACCCGCCGAAGCGGGTTTTTACGTACTCCAAGCTTGCCGGCTCTAGACGCGAGCTCG	Constructing pHMB1
pHMB2 oligo1	GAGCGGATAACAATATAATGAACCCGGGCTCGCGTCTAGAGCCGGCAAG	Constructing pHMB2
pHMB2 oligo2	CTTGCCGGCTCTAGACGCGAGCCCGGGTTCATTATATTGTTATCCGCTC	Constructing pHMB2
rnpB Xb1	ACTCCAAGCTTGCCGGCTCTAGA	Northern probe for modified rnpB T1
C0067Fw	GGC TGA ATT CAT AAG AGT TAG CTG T	C0067 cloning into pHMB1
C0067Rv	GGC TTC TAG AGA GTT AAA CCT CTA T	C0067 cloning into pHMB1
C0362Fw	GGC TGA ATT CAA GGG ATA TAA TCT C	C0362 cloning into pHMB1
C0362Rv	GGC TTC TAG AGT GTC GCG ATT ACG C	C0362 cloning into pHMB1
C0664Fw	GGC TGA ATT CAT GCC GAT GTA GGT G	C0664 cloning into pHMB1
C0664Rv	GGC TTC TAG ACG CGT TGG TTA GCG G	C0664 cloning into pHMB1
C0719Fw	GGC TGA ATT CAA GAG GAC TGA ACT G	C0719 cloning into pHMB1
C0719Rv	GGC TTC TAG ACC CCG GCA AGT TTT A	C0719 cloning into pHMB1
CsrBFw	GGC TGA ATT CAG TCG ACA GGG AGT C	CsrB cloning into pHMB1
CsrBRv	GGC TTC TAG AAA AAA CTG CCG CGA A	CsrB cloning into pHMB1
CsrCFw	GGC TGA ATT CAT AGA GCG AGG ACG C	CsrC cloning into pHMB1
CsrCRv	GGC TTC TAG AGG CGG AAT CTA ACA G	CsrC cloning into pHMB1
IS128Fw	AAG AGA GTA ATT ATT GTT TAT	IS128 cloning into pHMB2
IS128Rv	GGC TTC TAG ATG CAT TAC TAC GGT A	IS128 cloning into pHMB2
McaSFw	GGC TGA ATT CAC GAT ATT AAT AAT G	McaS cloning into pHMB1
McaSRv	GGC TTC TAG AAA TGC GGC TAT CTG C	McaS cloning into pHMB1
IsrBFw	GGC TGA ATT CAT GTC ATA TTA TAA G	IsrB cloning into pHMB1
IsrBRv	GGC TTC TAG AGA CTT CAC ATC TTC A	IsrB cloning into pHMB1
IsrCFw	GGC TGA ATT CAT AGC TCA ATA ATA G	IsrC cloning into pHMB1
IsrCRv	GGC TTC TAG ATT TCA TCA GCT TTT C	IsrC cloning into pHMB1
IstR2Fw	GGC TGA ATT CAG CAC TAA ATA CGT C	IstR2 cloning into pHMB1
IstR2Rv	GGC TTC TAG ACG TTT TGT ACT GAA T	IstR2 cloning into pHMB1
pRNAFw	AAT TCA TCG GCT CAG GGG ACT GGC CT	pRNA cloning into pHMB1
pRNARv	CTA GAG GCC AGT CCC CTG AGC CGA TG	pRNA cloning into pHMB1
PsrDFw	GGC TGA ATT CAT AGG CAT ATT TTT T	PsrD cloning into pHMB1
PsrDRv	GGC TTC TAG AAT TAA CTT TGT AAC G	PsrD cloning into pHMB1
RnpBFw	GGC TGA ATT CGA AGC TGA CCA GAC A	RnpB cloning into pHMB1
RnpBRv	GGC TTC TAG ACA AAA GCA AAA ACC C	RnpB cloning into pHMB1
RttRFw	GGC TGA ATT CAT CCC CCA CCA CCA T	RttR cloning into pHMB1
RttRRv	GGC TTC TAG ATT GCG GAT TCG CTT G	RttR cloning into pHMB1
RybAFw	GGC TGA ATT CAG CCT GTG CTA TAT C	RybA cloning into pHMB1
RybARv	GGC TTC TAG ATT TTC AGA CCA AAA C	RybA cloning into pHMB1
RydBFw	GGC TGA ATT CAC TAA TCG CAG TTT G	RydB cloning into pHMB1
RydBRv	GGC TTC TAG AAG AAA TAA GAA AAC C	RydB cloning into pHMB1
RyeFFw	GGC TGA ATT CAT TTT TAC CGT TGC A	RyeF cloning into pHMB1
RyeFRv	GGC TTC TAG AAG ATT CCT CTA ATT A	RyeF cloning into pHMB1
RyfAFw	GGC TGA ATT CAG CGG CCC TTT CCG C	RyfA cloning into pHMB1
RyfARv	GGC TTC TAG AGA CTT TAA ACA GAA T	RyfA cloning into pHMB1
RyfBFw	ACG TTA TTG AAG ATT TTG CTG	RyfB cloning into pHMB2
RyfBRv	GGC TTC TAG AAA GTT AAT TTA TAT A	RyfB cloning into pHMB2
RyfDFw	GGC TGA ATT CAA TCA AGA CGA TCC G	RyfD cloning into pHMB1
RyfDRv	GGC TTC TAG AAA GTG ATT GGG CAT C	RyfD cloning into pHMB1

SroCFw	GGC TGA ATT CAA GGC ACT GAA CTA A	SroC cloning into pHMB1
SroCRv	GGC TTC TAG ACA GTC TAT AGA CAT A	SroC cloning into pHMB1
SroEFw	GGC TGA ATT CAG TTC AGC AGG TTG A	SroE cloning into pHMB1
SroERv	GGC TTC TAG ATG CCA CGT TTA TTC T	SroE cloning into pHMB1
SroGFw	GGC TGA ATT CAG CTT ATT CTC AGG G	SroG cloning into pHMB1
SroGRv	GGC TTC TAG AAT AGC CAA AAT AAC G	SroG cloning into pHMB1
SroHFw	GGC TGA ATT CAG AAA ATA AGA ACA C	SroH cloning into pHMB1
SroHRv	GGC TTC TAG ACA GCT TCC TCA GCC C	SroH cloning into pHMB1
SsrAFw	GGC TGA ATT CAG GGG CTG ATT CTG G	SsrA cloning into pHMB1
SsrARv	GGC TTC TAG ATC TGG TAA TCA CCG A	SsrA cloning into pHMB1
SsrSFw	GGC TGA ATT CAG AAG ACA AAA TTT C	SsrS cloning into pHMB1
SsrSRv	GGC TTC TAG ATC ATG GCT GGT GCC A	SsrS cloning into pHMB1
TffFw	GGC TGA ATT CAT TTG TGG TAT AAA G	Tff cloning into pHMB1
TffRv	GGC TTC TAG AGA AAC AGT TGC CAT G	Tff cloning into pHMB1
Tp2Fw	GGC TGA ATT CAT AAT TTT TAC CAG A	Tp2 cloning into pHMB1
Tp2Rv	GGC TTC TAG ATC GCG TCG CGA GAT G	Tp2 cloning into pHMB1
Tpke70Fw	GGC TGA ATT CAA GGA AAA AAC CTT A	Tpke70 cloning into pHMB1
Tpke70Rv	GGC TTC TAG AGC GCT CTA ACA AAG C	Tpke70 cloning into pHMB1
DicFER1Fw	GGC TGA ATT CAT TTC TGG TGA CGT T	DicF cloning into pHMB1
DicFXb1Rv	GGC TTC TAG AGC GCT CAG CCG CAT T	DicF cloning into pHMB1
DsrAER1Fw	GGC TGA ATT CAA CAC ATC AGA TTT	DsrA cloning into pHMB1
DsrAXb1Rv	GGC TTC TAG ATG AAG TGA ATC GTT G	DsrA cloning into pHMB1
FnrSER1Fw	GGC TGA ATT CAG CAG GTG AAT GCA A	FnrS cloning into pHMB1
FnrSXb1Rv	GGC TTC TAG AGG GTA GAC GCT GAT A	FnrS cloning into pHMB1
GadYER1Fw	GGC TGA ATT CAC TGA GAG CAC AAA G	GadY cloning into pHMB1
GadYXb1Rv	GGC TTC TAG AGA TTA TAG AGT TTT A	GadY cloning into pHMB1
GcvBER1Fw	GGC TGA ATT CAC TTC CTG AGC CGG	GcvB cloning into pHMB1
GcvBXb1Rv	GGC TTC TAG AGA TCA GTA ATT CGC G	GcvB cloning into pHMB1
IS118ER1Fw	GGC TGA ATT CAG GTT CTG GAG GGG	IS118 cloning into pHMB1
IS118Xb1Rv	GGC TTC TAG ATT AAT TTA TAA AAT A	IS118 cloning into pHMB1
MgrRER1Fw	GGC TGA ATT CAG ATT CGT TAT CAG T	MgrR cloning into pHMB1
MgrRXb1Rv	GGC TTC TAG AAA GGG GCC TGA TTT T	MgrR cloning into pHMB1
MicAbluntFw	AGA AAG ACG CGC ATT TGT T	MicA cloning into pHMB2
MicAXb1Rv	GGC TTC TAG AGT GGC TGG AAA AAC A	MicA cloning into pHMB2
MicCER1Fw	GGC TGA ATT CAG TTA TAT GCC TTT A	MicC cloning into pHMB1
MicCXb1Rv	GGC TTC TAG ACT CTG GAT AAG GAT T	MicC cloning into pHMB1
MicFER1Fw	GGC TGA ATT CAG CTA TCA TCA TTA A	MicF cloning into pHMB1
MicFXb1Rv	GGC TTC TAG ATA ATG AAA AGT GTG T	MicF cloning into pHMB1
MicMER1Fw	GGC TGA ATT CAC ACC GTC GCT TAA A	MicM cloning into pHMB1
MicMXb1Rv	GGC TTC TAG AGG GAA TTT GCC GCA A	MicM cloning into pHMB1
OmrABER1Fw	GGC TGA ATT CAC CCA GAG GTA TTG AT	OmrAB cloning into pHMB1
OmrAXb1Rv	GGC TTC TAG ACG CGA GCG ACA GTA A	OmrA cloning into pHMB1
OmrBXb1Rv	GGC TTC TAG ATT ACT GTT ACA GAT T	OmrB cloning into pHMB1
OxySER1Fw	GGC TGA ATT CAG AAA CGG AGC GGC A	OxyS cloning into pHMB1
OxySXb1Rv	GGC TTC TAG AGC TTA TCG CCG GGC T	OxyS cloning into pHMB1
RprAER1Fw	GGC TGA ATT CAC GGT TAT AAA TCA A	RprA cloning into pHMB1
RprAXb1Rv	GGC TTC TAG ACG AGG TAG CGA AGC G	RprA cloning into pHMB1
RseXER1Fw	GGC TGA ATT CAT TTT TAT TAT TCT GT	RseX cloning into pHMB1
RseXXb1Rv	GGC TTC TAG AAT CAG GCG CAC ATT A	RseX cloning into pHMB1
RybBER1Fw	GGC TGA ATT CAC TGC TTT TCT TTG A	RybB cloning into pHMB1
RybBXb1Rv	GGC TTC TAG AGC AGG GTA GTA GAT A	RybB cloning into pHMB1
RybDER1Fw	GGC TGA ATT CAT ATC TGT AAT AAG	RybD cloning into pHMB1
RybDXb1Rv	GGC TTC TAG ACA GCG AAA ATC ATC G	RybD cloning into pHMB1
RydCER1Fw	GGC TGA ATT CAC TTC CGA TGT AGA C	RydC cloning into pHMB1
RydCXb1Rv	GGC TTC TAG ACG TAA ACG TTC CTG A	RydC cloning into pHMB1
SpfER1Fw	GGC TGA ATT CAG TAG GGT ACA GAG G	Spf cloning into pHMB1
SpfXb1Rv	GGC TTC TAG AGC GCA TCA GGC ATT A	Spf cloning into pHMB1
SgrSER1Fw	GGC TGA ATT CAG ATG AAG CAA GGG G	SgrS cloning into pHMB1
SgrSXb1Rv	GGC TTC TAG AGT TCC CTT TTT AGC G	SgrS cloning into pHMB1

ArcZER1Fw	GGCCGAATTCGTGCGGCCTGAAAAAC	ArcZ cloning into pHM-tac
ArcZXb1Rv	GGC TTC TAG AGA AAG CGT GGG TGG C	ArcZ cloning into pHM-tac
GlmYER1Fw	CGGAATTC AGT GGC TCA TTC ACC GAC	GlmY cloning into pHM-tac
GlmYXb1Rv	GCTCTAGA CAGTTGTCGT AATGAGCG	GlmY cloning into pHM-tac
GlmZER1Fw	CGGAATTC GTA GAT GCT CAT TCC ATC	GlmZ cloning into pHM-tac
GlmZXb1Rv	GCTCTAGA AATAACCCGC CACAGTAG	GlmZ cloning into pHM-tac
PsrOER1Fw	CGGAATTC ATC TTC TGC GCA TCC TCG	PsrO cloning into pHM-tac
PsrOXb1Rv	GCTCTAGA CGTCGTAAAC TGCTCGAC	PsrO cloning into pHM-tac
RyhBER1Fw	CGGAATTC GCG ATC AGG AAG ACC C	RyhB cloning into pHM-tac
RyhBXb1Rv	GCTCTAGA GTTATTTACA CCTTAGCGC	RyhB cloning into pHM-tac
SdsRER1Fw	CGGAATTC GG CAAGGCAACT AAGC	SdsR cloning into pHM-tac
SdsRXb1Rv	GCTCTAGA ATAAAGTCAG CGAAGG	SdsR cloning into pHM-tac
RyeAER1Fw	CGGAATTC AAAGTCA GCGAAGGAAA TGC	RyeA cloning into pHM-tac
RyeAXb1Rv	GCTCTAGA CTGAAAAACT CTGTTAATTA CC	RyeA cloning into pHM-tac
CyaRER1Fw	CCG GAA TTC GCT GAA AAA CAT AAC	CyaR cloning into pHM-tac
CyaRXb1Rv	TGC TCT AGA TAG AAA CAG GAA GCC	CyaR cloning into pHM-tac
Och1Fw	GGCT GAATTC ATG CCT TTG GTA GAG GAA	Och1 cloning into pHMB1
Och1Rv	GGCT TCTAGA CGC TCT TTA ATT GTT AAT	Och1 cloning into pHMB1
Och2Fw	GGC TGA ATT CAT GCG CAT CAG TAA TAA A	Och2 cloning into pHMB1
Och2Rv	GGCT TCTAGA CGC GTT TGC ATC GTC GTC	Och2 cloning into pHMB1
Och3Fw	GCT GAA TTC ATA TAT AAT TTA TAT TAT TC	Och3 cloning into pHMB1
Och3Rv	GGCT TCTAGA ATT TGC CGA CCC CGA GCA	Och3 cloning into pHMB1
Och4Fw	GGC TGA ATT CAC GAT ATA TGT TTA TTT T	Och4 cloning into pHMB1
Och4Rv	GGCT TCTAGA TTG TAA TGA AGT CAC TTA	Och4 cloning into pHMB1
Och5Fw	GGC TGA ATT CAT CCT TGT CCG CTT AAC C	Och5 cloning into pHMB1
Och5Rv	GGCT TCTAGA CAA CCC GCT AAA ACA CCT	Och5 cloning into pHMB1
Och6Fw	GGC TGA ATT CAG CGC CTT GAA AAG AGG C	Och6 cloning into pHMB1
Och6Rv	GGCT TCTAGA TAA GCA AAA GTG ATT TAT	Och6 cloning into pHMB1
Och7Fw	GGC TGA ATT CAG GTG AGC ACT GAA CAA T	Och7 cloning into pHMB1
Och7Rv	GGCT TCTAGA GAA TGA TTT AGG CGG TTC	Och7 cloning into pHMB1
Och8Fw	GGCT GAATTC ACA CGA TCA CTC TAA GAG	Och8 cloning into pHMB1
Och8Rv	GGCT TCTAGA AGA CCA TTA AAC AGC CTG	Och8 cloning into pHMB1
Och9Fw	GGC TGA ATT CAC CAT CGT GCA AAA GGG C	Och9 cloning into pHMB1
Och9Rv	GGCT TCTAGA TCC AAA GGT CAT TGC ACC	Och9 cloning into pHMB1
Och10Fw	GGCT GAATTC ATA CTA GCC ACT GAA AAT	Och10 cloning into pHMB1
Och10Rv	GGCT TCTAGA AGC AGA GTA TGA CTA AAA	Och10 cloning into pHMB1
C0343_NP	GTC AGG CAT GGG TTG CGG GCG AGG	Northern probe for C0343
C0362_NP	GAC AAA CGC AAC ATT GCC TGA TGC	Northern probe for C0362
CsrB_NP	GCC TTT CCC TGA AAC ACC ATC CTG G	Northern probe for CsrB
CsrC_NP	GAT TCC ATT TCC GTT TAA TTA CGT CT	Northern probe for CsrC
CyaR_NP	GGT TCC TGG TAC AGC TAG CAT TTT ATG	Northern probe for CyaR
DsrA_NP	GTT ACA CCA GGA AAT CTG ATG TGT T	Northern probe for DsrA
FnrS_NP	GGA AGT AAG ACA ATA TGG AGC GCA A	Northern probe for FnrS
GadY_NP	GAG GAT AGT CTG CCG TCT CCA GAC TAA TAA	Northern probe for GadY
GcvB_NP	GCA TTC CGA TAA AAC TTT TCG TTC C	Northern probe for GcvB
IsrB_NP	GAA CAT ACT TTT CAG GAT TTT GCG C	Northern probe for IsrB
IstR-1_NP	GCA AAG CAC ACT GTA TTA TGT CAA C	Northern probe for IstR-1
IstR-2_NP	GCA CGA ATT TTG ACG TAT TTA GTG C	Northern probe for IstR-2
MgrR_NP	GTA AAC CGG CGG TGA ATG CTT GCA T	Northern probe for MgrR
OmrA_NP	AGA GCG TAC CGA ATA ATC TCA CCA A	Northern probe for OmrA
OmrB_NP	GCT CAA CCC GAA GTT GAC TTC ACC T	Northern probe for OmrB
OxyS_NP	GAA ACG GGC AGT GAC TTC AAG GGT	Northern probe for OxyS
PsrO_NP	GCT GAC GGC AGC AAT TCA CTG GAA A	Northern probe for PsrO
RseX_NP	GAT AAA AGG CTA ATA ACG GAA GCA T	Northern probe for RseX
RttR_NP	GCA GAA TAT TTA ATT GCG GAT TCG T	Northern probe for RttR
RybB_NP	ATG GGG ACA TCA AAG AAA AGC AGT	Northern probe for RybB
SdsR_NP	GGC TCT TGG GAG AGA GCC GTC G	Northern probe for SdsR
RyeF_NP	GAA TAT GAT TTA TTA CAA TGT AAT CAT T	Northern probe for RyeF
RyhB_NP	GCA ATG TCG TGC TTT CAG GTT CTC	Northern probe for RyhB

SokX_NP	TGA GGC CAG CGT TCG AAC CCA AA	Northern probe for SokX
Spf_NP	GAA GTA AAA GGT CTG AAA GAT AGA A	Northern probe for Spf
Och10_NP	GAT CCT CGG AAG GGG AAG TTG ATT A	Northern probe for Och10
ΔarcZFw	TACGCATCACACATTTAACTGATTCATGTAACAAAT CATTTAAGTTGTAGGCTGGAGCTGCTTCG	*arcZ* knockout
ΔarcZRv	GCTAGACCGGGGTGCGCGAATACTGCGCCAACAC CAGGGAAATCTTTCCGGGGATCCGTCGACC	*arcZ* knockout
ΔcsrBFw	TGTAAGCGCCTTGTAAGACTTCGCGAAAAAGACGA TTCTATCTTCTGTAGGCTGGAGCTGCTTCG	*csrB* knockout
ΔcsrBRv	GTATTCACAGCGCTCCCGGTTCGTTTCGCAGCATT CCAGCTACTTTTCCGGGGATCCGTCGACC	*csrB* knockout
ΔcsrCFw	TGGCGGTTGATTGTTTGTTTAAAGCAAAGGCGTAA AGTAGCACCCTGTAGGCTGGAGCTGCTTCG	*csrC* knockout
ΔcsrCRv	GATTTGCGGCGGAATCTAACAGAAAGCAAGCAAA GAAAAAAGGCGTTCCGGGGATCCGTCGACC	*csrC* knockout
ΔcyaRFw	AACCGATCACATACAGCTGCATTTATTAAGGTTAT CATCCGTTTCTGTAGGCTGGAGCTGCTTCG	*cyaR* knockout
ΔcyaRRv	GGACGTGACCAGAAATAAATCCTTTTATTTCATTGT ATTACGCGTTTCCGGGGATCCGTCGACC	*cyaR* knockout
ΔdicFFw	ATACGCTTAAGTGACAACCCCGCTGCAACGCCCT CTGTTATCAATTGTAGGCTGGAGCTGCTTCG	*dicF* knockout
ΔdicFRv	GTGCGCTCAGCCGCATTCACCACATCACAAAATTC ACTTTAAAAATTCCGGGGATCCGTCGACC	*dicF* knockout
ΔdsrAFw	GGGTGACGTGCGTCACATTTCTATTCATAAGTAGC GTTAATCATTTCCGGGGATCCGTCGACC	*dsrA* knockout
ΔdsrARv	ATAAAAAAATCCCGACCCTGAGGGGGTCGGGATG AAACTTGCTTATGTAGGCTGGAGCTGCTTCG	*dsrA* knockout
ΔfnrSFw	GCGAAGTCAATAAACTCTCTACCCATTCAGGGCAA TATCTCTCTTTGTAGGCTGGAGCTGCTTCG	*fnrS* knockout
ΔfnrSRv	TCATTGGGTGGACTCTTAAAGGGTAGACGCTGATA AATAACAGGCTTCCGGGGATCCGTCGACC	*fnrS* knockout
ΔgadYFw	AAAAAAATGGCTGATCTTATTTCCAGTAAAAGTTAT ATTTAACTTTGTAGGCTGGAGCTGCTTCG	*gadY* knockout
ΔgadYRv	AGGGGACCGGGAAGAGGATAGTCTGCCGTCTCCA GACTAATAAACTTCCGGGGATCCGTCGACC	*gadY* knockout
ΔgcvBFw	AAATTGTCCGTTGAGCTTCTACCAGCAAATACCTA TAGTGGCGGCTGTAGGCTGGAGCTGCTTCG	*gcvB* knockout
ΔgcvBRv	GATCGCAAGGTAAAAAAAAGCACCGCAATTAGGC GGTGCTACATTTTCCGGGGATCCGTCGACC	*gcvB* knockout
ΔglmYFw	CCAAACTATTTTCTTTATTGGCACAGTTACTGCATA ATAGTAACCTGTAGGCTGGAGCTGCTTCG	*glmY* knockout
ΔglmYRv	GAATAGCCTGATGCTAACCGAGGGGAAGTTCAGA TACAACAAAGCTTCCGGGGATCCGTCGACC	*glmY* knockout
ΔIS118Fw	AGTTAGTATATGTATCTATCACTGTTGATGATAATA TCAGCACTTTGTAGGCTGGAGCTGCTTCG	*IS118* knockout
ΔIS118Rv	AATTGCGCGCCAATCATGGCGCGCACAAGCTATA ATACCAACCTATTCCGGGGATCCGTCGACC	*IS118* knockout
ΔmcaSFw	TGAAGAAAATTGGCAACTAAAGGTTAAAACCGTTA TAACACAGTCTGTAGGCTGGAGCTGCTTCG	*mcaS* knockout
ΔmcaSRv	CATAAAAAAATAGAGTCTGTCGACATCCGCCAGAC TCTACAGTACTTCCGGGGATCCGTCGACC	*mcaS* knockout
ΔmicAFw	TAAAAATTTTCTGAACTCTTTCTTCCCAGGCGAGTC TGAGTATATTGTAGGCTGGAGCTGCTTCG	*micA* knockout
ΔmicARv	AAGAAAAAGGCCACTCGTGAGTGGCCAAAATTTCA TCTCTGAATTTTCCGGGGATCCGTCGACC	*micA* knockout
ΔmicCFw	AAATAAAAATTATACTTTTAATTTGCTATACGTTATT CTGCGCGGTGTAGGCTGGAGCTGCTTCG	*micC* knockout
ΔmicCRv	CTGGATAAGGATTATCCAATTCTAAAAAAAAAGCC CGGACGACTGTTCCGGGGATCCGTCGACC	*micC* knockout

ΔmicMFw	CCTAAGAGTATTGGCAGGATGGTGAGATTGAGCG ACAATCGAGTTTGTAGGCTGGAGCTGCTTCG	*micM* knockout
ΔmicMRv	TGGCCAATATCGCTATTGGCCCGTCAAAGAGGAAT TTCATTTTTTTTCCGGGGATCCGTCGACC	*micM* knockout
ΔomrAFw	GTTTTCTCGCTGGCGAAGAGTCGTCGTGCAGACC ACAATCAAGATTGTAGGCTGGAGCTGCTTCG	*omrA* knockout
ΔomrARv	TAAAGAACGCGAGCGACAGTAAATTAGGTGCGAA AAAAAACCTGCTTCCGGGGATCCGTCGACC	*omrA* knockout
ΔomrBFw	ATTGACCGCTGGTGGCGTTTGGCTTCAGGTTGCT AAAGTGGTGATTGTAGGCTGGAGCTGCTTCG	*omrB* knockout
ΔomrBRv	CGAGGTGTGTAAATTGTCGGTTACTGTTACAGATT GATGACCGGCTTCCGGGGATCCGTCGACC	*omrB* knockout
ΔoxySFw	TGAACGATTATCCCTATCAAGCATTCTGACTGATA ATTGCTCACATGTAGGCTGGAGCTGCTTCG	*oxyS* knockout
ΔoxySRv	AAATTTGAGCCTGGCTTATCGCCGGGCTTTTTTAT GGCAAAAAAATTCCGGGGATCCGTCGACC	*oxyS* knockout
ΔrdlBFw	CGAGCGTCATATAGCCGCCTTGTTGTAATGACAAC ATTTTGCGGCTGTAGGCTGGAGCTGCTTCG	*rdlB* knockout
ΔrdlBRv	AATGTTGCGGGGGCTTTATCCCTGGTGGCATTGG TTGCTGGAAAGTTCCGGGGATCCGTCGACC	*rdlB* knockout
ΔrdlCFw	CGAGCGTCATATAGCCGCCTTGTTGTAATGACAAC ATTTTGCGGCTGTAGGCTGGAGCTGCTTCG	*rdlC* knockout
ΔrdlCRv	CGCAATGTTGCGGGGCTTTATCCCTGGTGGCATT GGTTGCTGGAATTCCGGGGATCCGTCGACC	*rdlC* knockout
ΔrprAFw	CATTCAGCTGGTAGTACCTGTCGCAAATTCTTTAC AGTTTTTTGTAGGCTGGAGCTGCTTCG	*rprA* knockout
ΔrprARv	TCAGCCTGCTGACGGCTTGAAGAGAGTCACAGTA TCTTGTGCAACTTCCGGGGATCCGTCGACC	*rprA* knockout
ΔrseXFw	ATTAATTCATTTAATCAATATATTAGCACTGATTACA ATTATACCTGTAGGCTGGAGCTGCTTCG	*rseX* knockout
ΔrseXRv	TGCGCCAAACGGCTGGTGTGATCAGGCGCACATT AATGAAGGCATTTCCGGGGATCCGTCGACC	*rseX* knockout
ΔrybBFw	ACAACCGCAGAACTTTTCCGCAGGGCATCAGTCTT AATTAGTGCCTGTAGGCTGGAGCTGCTTCG	*rybB* knockout
ΔrybBRv	AAGTTTTAGATAACAAAAAACCCATCAACCTTGAAC CGAAATGGCTTCCGGGGATCCGTCGACC	*rybB* knockout
ΔrydCFw	CAAGGATTATGGTTTTATTTATCATACAAATAAATA TAATAGGCGTGTAGGCTGGAGCTGCTTCG	*rydC* knockout
ΔrydCRv	ATTTAAAGAAAACGCCTGTACTAAAACCGACCCGT GGTACAGGCGTTCCGGGGATCCGTCGACC	*rydC* knockout
ΔsdsRFw	AAAACGCTGGACTCAGACAGTAGAGTGTGTGTTAT GGTTGACTATTGTAGGCTGGAGCTGCTTCG	*sdsR* knockout
ΔsdsRRv	TAAAATAGCCTTTTGATTTCCAATAAAAAAACCGCC TCAGTTCTTTTCCGGGGATCCGTCGACC	*sdsR* knockout
ΔryeFFw	GAAATGAAAGGAATCATTGAACGCCATCAGGCCAA ATGATTTTTATGTAGGCTGGAGCTGCTTCG	*ryeF* knockout
ΔryeFRv	AAAAACAATAACCCAATGCGTTTTTTCCCTCGCATA GATTCCTCTTTCCGGGGATCCGTCGACC	*ryeF* knockout
ΔryfAFw	CAAGCAAAAGAGAGTTATTATTGTTCTGTTAGTGTA TTATCCACTTGTAGGCTGGAGCTGCTTCG	*ryfA* knockout
ΔryfARv	GCCGCCCAAAGGGCGGCATTCGGGCTTACTTGCT GCTCTGAAGGTTTCCGGGGATCCGTCGACC	*ryfA* knockout
ΔryfBFw	AAGATTAACGTGACCGCCAATTCGTAAGTACATTA AAATTGGCTTTGTAGGCTGGAGCTGCTTCG	*ryfB* knockout
ΔryfBRv	TCACTAAGTTAATTTATATAGTATCTGCCCAGACAC TTATTTATATTCCGGGGATCCGTCGACC	*ryfB* knockout
ΔryfDFw	CCACCGGTCGTATGGCAAGTTTCATTTGGCTGATA TAACCTAAAGTGTAGGCTGGAGCTGCTTCG	*ryfD* knockout
ΔryfDRv	CTACTGGAGATTAAATGAGGTCATCCCTCAATTAT TCAAGGTTATTTCCGGGGATCCGTCGACC	*ryfD* knockout

ΔryhBFw	AAAAAGTGTTGGACAAGTGCGAATGAGAATGATTATTATTGTCTCTGTAGGCTGGAGCTGCTTCG	*ryhB* knockout
ΔryhBRv	AACACAAGCACTCCCGTGGATAAATTGAGAACGAAAGATCAAAAATTCCGGGGATCCGTCGACC	*ryhB* knockout
ΔsgrSFw	TTCCCTATATTAAGTCAATAATTCCTAACGATGAAGCAAGGGGGTTGTAGGCTGGAGCTGCTTCG	*sgrS* knockout
ΔsgrSRv	CGTCATTATCCAGATCATACGTTCCCTTTTTAGCGCGGCGAGAATTTCCGGGGATCCGTCGACC	*sgrS* knockout
ΔsroCFw	GCACTGTTCAAAGAACCGAATGACAAGGCACTGAACTAATTACAATGTAGGCTGGAGCTGCTTCG	*sroC* knockout
ΔsroCRv	GTAAAAAAATACCCCAGTTCCAGTCTATAGACATAAATCTACTCCTTCCGGGGATCCGTCGACC	*sroC* knockout
Δoch5Fw	TTGCGCATCTTTTTCGAAATAAAAATGTCCATCCCCCTCCCCCGCTGTAGGCTGGAGCTGCTTCG	*och5* knockout
Δoch5Rv	GTAGCCCGACGTATTCGTCGGGCTAATGCCTTTACCCGATATTGCTTCCGGGGATCCGTCGACC	*och5* knockout
T7-csgDFw	TAATACGACTCACTATAGGGCAGATGTAATCCATTAGTTT	*csgD* 5'-UTR *in vitro* transcription
csgDRv	GACTTCATTAAACATGATGAAACCC	*csgD* 5'-UTR *in vitro* transcription
T7-flhDFw	TAATACGACTCACTATAGGGGATTTAGGAAAAATCTTAGA	*flhD* 5'-UTR *in vitro* transcription
flhDRv	CTCGGAGGTATGCATTATTCCCACC	*flhD* 5'-UTR *in vitro* transcription
T7-och5Fw	TAATACGACTCACTATAGGGATCCTTGTCCGCTTAACCGTT	Och5 *in vitro* transcription
och5Rv	AATAATATTTGTAGCCCGACGTATTC	Och5 *in vitro* transcription
T7-dicFFw	TAATACGACTCACTATAGGGTTTCTGGTGACGTTTGGCGG	DicF *in vitro* transcription
dicF5Rv	GGGCGGCAGAGCAGTC	DicF *in vitro* transcription
T7-omrAFw	TAATACGACTCACTATAGGGCCCAGAGGTATTGATTGGTG	OmrA *in vitro* transcription
omrARv	AAAAAAAACCTGCGCATCCGC	OmrA *in vitro* transcription
T7-omrBFw	TAATACGACTCACTATAGGGCCCAGAGGTATTGATAGGTGA	OmrB *in vitro* transcription
omrBRv	AAAAAAAACCTGCGCATCTGCG	OmrB *in vitro* transcription
T7-rprAFw	TAATACGACTCACTATAGGGACGGTTATAAATCAACATATTGATTTATAAG	RprA *in vitro* transcription
rprARv	AAAAAAAGCCCATCGTGGGAG	RprA *in vitro* transcription
T7-ssrSFw	TAATACGACTCACTATAGGGATTTCTCTGAGATGTTCGCAAGC	6S *in vitro* transcription
ssrSRv	GAATCTCCGAGATGCCGC	6S *in vitro* transcription
T7-sdsRFw	TAATACGACTCACTATAGGGCAAGGCAACTAAGCCTGCAT	SdsR *in vitro* transcription
sdsRRv	AAAAAGAGACCGAACACGATTCCTG	SdsR *in vitro* transcription
T7-IS118Fw	TAATACGACTCACTATAGGGGGTTCTGGAGGGGGTTTG	IS118 *in vitro* transcription
IS118Rv	AAATTGCGCGCCAATCATGG	IS118 *in vitro* transcription
T7-sgrSFw	TAATACGACTCACTATAGGGGATGAAGCAAGGGGGTGC	SgrS *in vitro* transcription
sgrSRv	AAAAAAAACCAGCAGGTATAATCTGC	SgrS *in vitro* transcription

Supplementary Figures

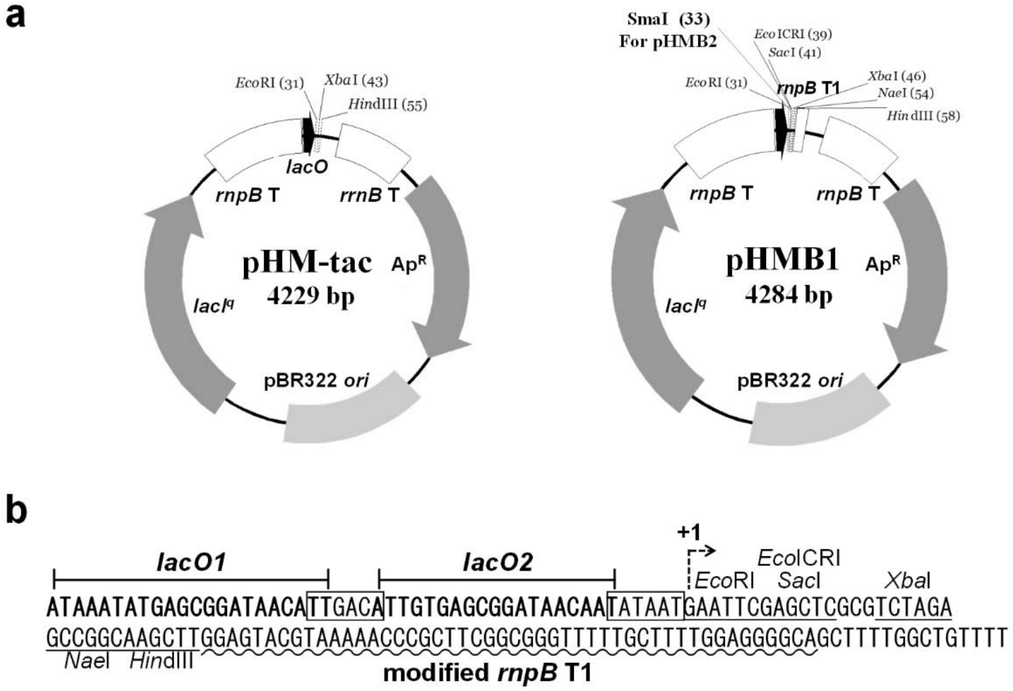

Supplementary Figure 1. sRNA expression vectors pHM-tac and pHMB1. (a) Schematic view of plasmids pHM-tac and pHMB1 containing the pBR322 replication origin, ampicillin resistance gene, and the tacO promoter, which is tightly controlled by the *lacI^q* gene on plasmids and induced by IPTG. pHMB1 was constructed by adding the modified *rnpB* T1 terminator in the region downstream of the multiple cloning site (MCS) of pHM-tac. pHMB2, a derivative of pHMB1, has the *Sma*I cloning site instead of *Eco*RI. (b) Schematic structure of the transcription unit region of the pHMB1 vector. Lac operator sequences are shown in bold, and designated *lacO1* and *lacO2*. The putative transcription start site is indicated with an arrow, and the -35 and -10 regions of the tac promoter with boxes. Unique recognition sites for each restriction enzyme in MCS are underlined.

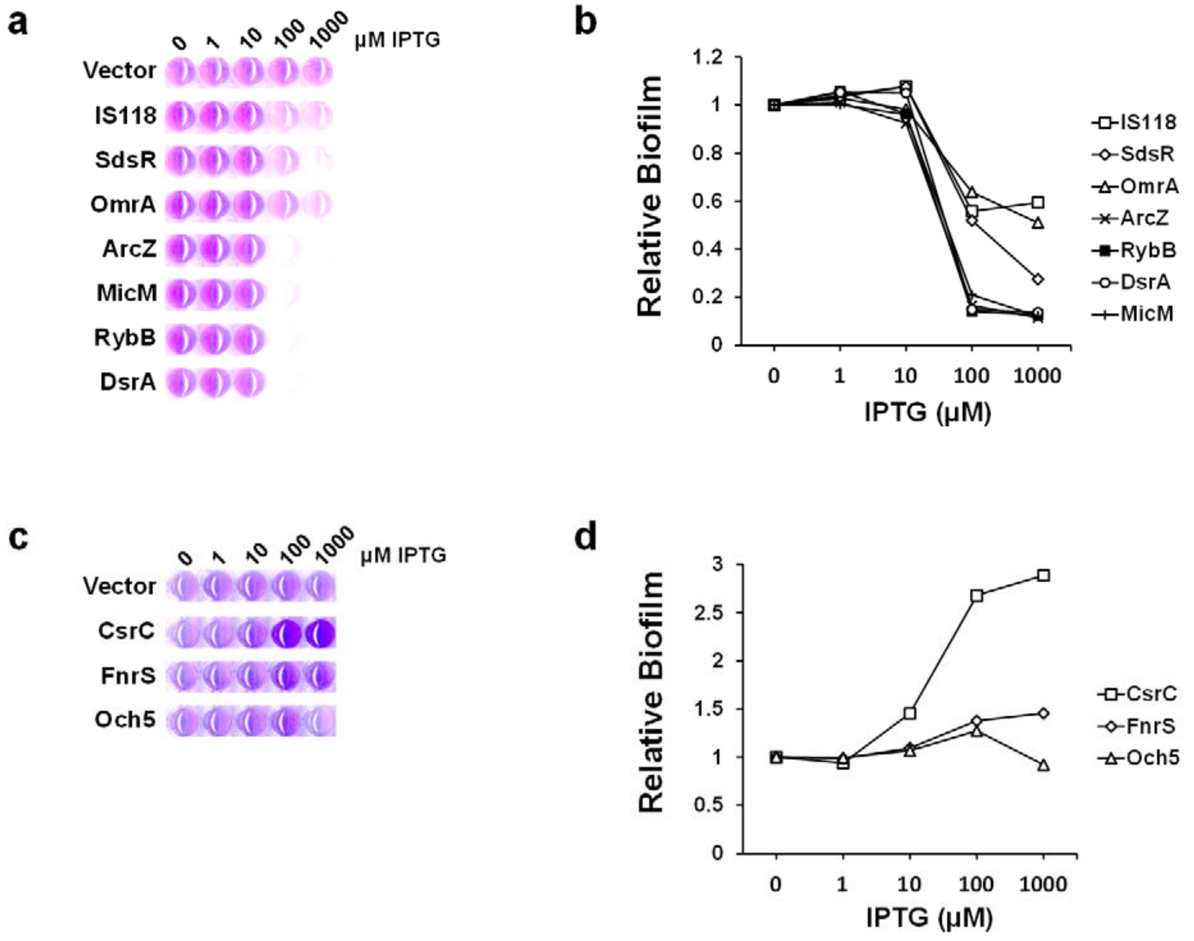

Supplementary Figure 2. Dosage effects of sRNAs on biofilm formation. (a, c) *E. coli* MG1655 strains containing each sRNA-expressing plasmid were grown in LB complemented with different IPTG concentrations at 30ºC for 12 h. The biofilm attached to 96-well round bottom polystyrene microtiter plates was determined by crystal violet staining and dissolved with 30% acetic acid. (b, d) The level of biofilm formation of each strain (OD_{550}/OD_{595}) was normalized to the level of the condition with no IPTG, and termed ‘relative biofilm’.

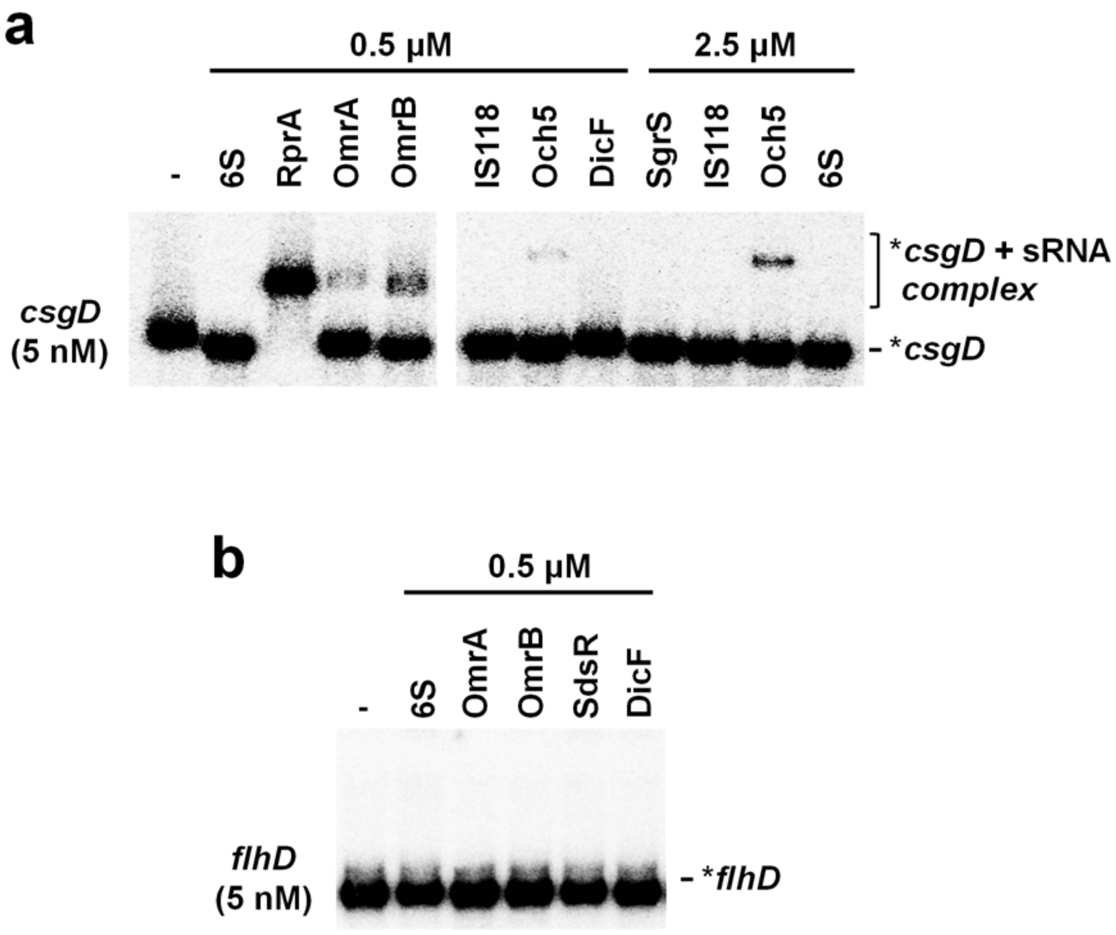

Supplementary Figure 3. Binding assay of sRNAs with *flhD* or *csgD* 5'-UTR RNA. ^{32}P-labeled 5'-UTR RNA (5 nM) was incubated at 25°C for 20 min with sRNAs (0.5 or 2.5 µM) in TMN binding buffer. After incubation, samples were separated on 5% non-denaturing polyacrylamide gels at 4°C. *csgD* 5'-UTR RNA (a) and *flhD* 5'-UTR RNA (b). 6S RNA was used as negative control. Asterisks indicate ^{32}P 5'-end labeled RNA species.

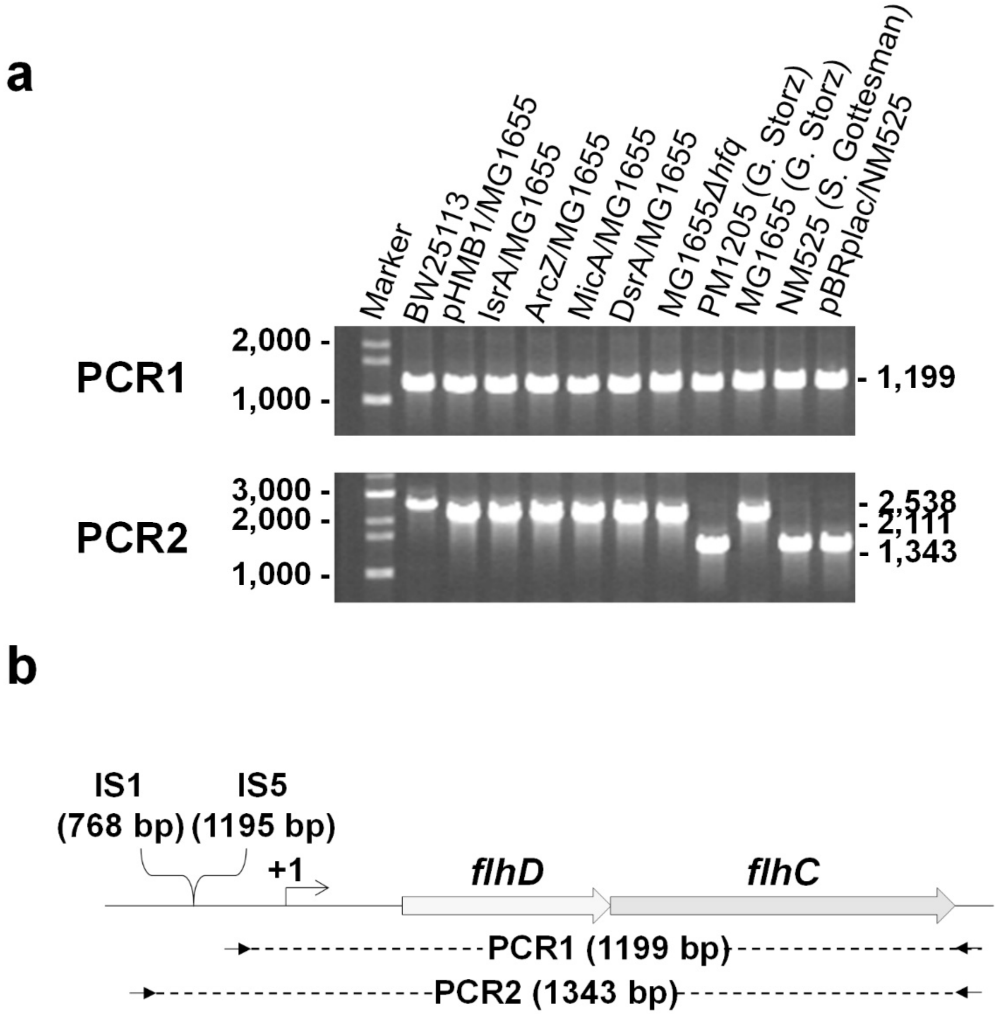

Supplementary Figure 4. PCR for detecting IS elements upstream of the *flhDC* operon. (a) Two amplified products were generated from chromosomal DNA of several *E. coli* strains. The 2,111 bp and 2,538 bp (bottom panel) products signify insertion of the IS1 or IS5 element, respectively. (b) Schematic view of the amplified region. The transcription start site of the *flhDC* operon is designated +1. Primer positions are indicated with arrows, and the expected PCR products (PCR1 and 2) with dashed lines.

Supplementary References

1. Argaman, L. *et al.* Novel small RNA-encoding genes in the intergenic regions of Escherichia coli. *Curr Biol* **11,** 941–950 (2001).

2. Wassarman, K. M., Repoila, F., Rosenow, C., Storz, G. & Gottesman, S. Identification of novel small RNAs using comparative genomics and microarrays. *Genes Dev* **15,** 1637–1651 (2001).

3. Papenfort, K. *et al.* Specific and pleiotropic patterns of mRNA regulation by ArcZ, a conserved, Hfq-dependent small RNA. *Mol Microbiol* **74,** 139–158 (2009).

4. Mandin, P. & Gottesman, S. Integrating anaerobic/aerobic sensing and the general stress response through the ArcZ small RNA. *EMBO J* **29,** 3094–3107 (2010).

5. De Lay, N. & Gottesman, S. A complex network of small non-coding RNAs regulate motility in Escherichia coli. *Mol Microbiol* (2012). doi:10.1111/j.1365-2958.2012.08209.x

6. Tjaden, B. *et al.* Transcriptome analysis of Escherichia coli using high-density oligonucleotide probe arrays. *Nucleic Acids Res* **30,** 3732–3738 (2002).

7. Rivas, E., Klein, R. J., Jones, T. A. & Eddy, S. R. Computational identification of noncoding RNAs in E. coli by comparative genomics. *Curr Biol* **11,** 1369–1373 (2001).

8. Liu, M. Y. *et al.* The RNA molecule CsrB binds to the global regulatory protein CsrA and antagonizes its activity in Escherichia coli. *J Biol Chem* **272,** 17502–17510 (1997).

9. Weilbacher, T. *et al.* A novel sRNA component of the carbon storage regulatory system of Escherichia coli. *Mol Microbiol* **48,** 657–670 (2003).

10. Johansen, J., Eriksen, M., Kallipolitis, B. & Valentin-Hansen, P. Down-regulation of outer membrane proteins by noncoding RNAs: unraveling the cAMP-CRP- and sigmaE-dependent CyaR-ompX regulatory case. *J Mol Biol* **383,** 1–9 (2008).

11. De Lay, N. & Gottesman, S. The Crp-activated small noncoding regulatory RNA CyaR (RyeE) links nutritional status to group behavior. *J Bacteriol* **191,** 461–476 (2009).

12. Bouche, F. & Bouche, J. P. Genetic evidence that DicF, a second division inhibitor encoded by the Escherichia coli dicB operon, is probably RNA. *Mol Microbiol* **3,** 991–994 (1989).

13. Faubladier, M., Cam, K. & Bouche, J. P. Escherichia coli cell division inhibitor DicF-RNA of the dicB operon. Evidence for its generation in vivo by transcription termination and by RNase III and RNase E-dependent processing. *J Mol Biol* **212,** 461–471 (1990).

14. Tetart, F. & Bouche, J. P. Regulation of the expression of the cell-cycle gene ftsZ by DicF antisense RNA. Division does not require a fixed number of FtsZ molecules. *Mol Microbiol* **6,** 615–620 (1992).

15. Sledjeski, D. & Gottesman, S. A small RNA acts as an antisilencer of the H-NS-silenced rcsA gene of Escherichia coli. *Proc Natl Acad Sci U S A* **92,** 2003–2007 (1995).

16. Sledjeski, D. D., Gupta, A. & Gottesman, S. The small RNA, DsrA, is essential for the low temperature expression of RpoS during exponential growth in Escherichia coli. *EMBO J* **15,** 3993–4000 (1996).

17. Majdalani, N., Cunning, C., Sledjeski, D., Elliott, T. & Gottesman, S. DsrA RNA regulates translation of RpoS message by an anti-antisense mechanism, independent of its action as an antisilencer of transcription. *Proc Natl Acad Sci U S A* **95,** 12462–12467 (1998).

18. Lease, R. A. & Belfort, M. A trans-acting RNA as a control switch in Escherichia coli: DsrA modulates function by forming alternative structures. *Proc Natl Acad Sci U S A* **97,** 9919–9924 (2000).

19. Saetrom, P. *et al.* Predicting non-coding RNA genes in Escherichia coli with boosted genetic programming. *Nucleic Acids Res* **33,** 3263–3270 (2005).

20. Ribes, V., Romisch, K., Giner, A., Dobberstein, B. & Tollervey, D. E. coli 4.5S RNA is part of a ribonucleoprotein particle that has properties related to signal recognition particle. *Cell* **63,** 591–600 (1990).

21. Poritz, M. A. *et al.* An E. coli ribonucleoprotein containing 4.5S RNA resembles mammalian signal recognition particle. *Science (80-.).* **250,** 1111–1117 (1990).

22. Durand, S. & Storz, G. Reprogramming of anaerobic metabolism by the FnrS small RNA. *Mol Microbiol* **75,** 1215–1231 (2010).

23. Boysen, A., Moller-Jensen, J., Kallipolitis, B., Valentin-Hansen, P. & Overgaard, M. Translational regulation of gene expression by an anaerobically induced small non-coding RNA in Escherichia coli. *J Biol Chem* **285,** 10690–10702 (2010).

24. Chen, S. *et al.* A bioinformatics based approach to discover small RNA genes in the Escherichia coli genome. *Biosystems* **65,** 157–177 (2002).

25. Opdyke, J. A., Kang, J. G. & Storz, G. GadY, a small-RNA regulator of acid response genes in Escherichia coli. *J Bacteriol* **186,** 6698–6705 (2004).

26. Tramonti, A., De Canio, M. & De Biase, D. GadX/GadW-dependent regulation of the Escherichia coli acid fitness island: transcriptional control at the gadY-gadW divergent promoters and identification of four novel 42 bp GadX/GadW-specific binding sites. *Mol Microbiol* **70,** 965–982 (2008).

27. Urbanowski, M. L., Stauffer, L. T. & Stauffer, G. V. The gcvB gene encodes a small untranslated RNA involved in expression of the dipeptide and oligopeptide transport systems in Escherichia coli. *Mol Microbiol* **37,** 856–868 (2000).

28. Sharma, C. M., Darfeuille, F., Plantinga, T. H. & Vogel, J. A small RNA regulates multiple ABC transporter mRNAs by targeting C/A-rich elements inside and upstream of ribosome-binding sites. *Genes Dev* **21,** 2804–2817 (2007).

29. Pulvermacher, S. C., Stauffer, L. T. & Stauffer, G. V. Role of the sRNA GcvB in regulation of cycA in Escherichia coli. *Microbiology* **155,** 106–114 (2009).

30. Pulvermacher, S. C., Stauffer, L. T. & Stauffer, G. V. The small RNA GcvB regulates sstT mRNA expression in Escherichia coli. *J Bacteriol* **191,** 238–248 (2009).

31. Sharma, C. M. *et al.* Pervasive post-transcriptional control of genes involved in amino acid metabolism by the Hfq-dependent GcvB small RNA. *Mol Microbiol* **81,** 1144–1165 (2011).

32. Jorgensen, M. G. *et al.* Small regulatory RNAs control the multi-cellular adhesive lifestyle of Escherichia coli. *Mol Microbiol* **84,** 36–50 (2012).

33. Urban, J. H., Papenfort, K., Thomsen, J., Schmitz, R. A. & Vogel, J. A conserved small RNA promotes discoordinate expression of the glmUS operon mRNA to activate GlmS synthesis. *J Mol Biol* **373,** 521–528 (2007).

34. Urban, J. H. & Vogel, J. Two seemingly homologous noncoding RNAs act hierarchically to activate glmS mRNA translation. *PLoS Biol* **6,** e64 (2008).

35. Reichenbach, B., Maes, A., Kalamorz, F., Hajnsdorf, E. & Gorke, B. The small RNA GlmY acts upstream of the sRNA GlmZ in the activation of glmS expression and is subject to regulation by polyadenylation in Escherichia coli. *Nucleic Acids Res* **36,** 2570–2580 (2008).

36. Göpel, Y., Papenfort, K., Reichenbach, B., Vogel, J. & Görke, B. Targeted decay of a regulatory small RNA by an adaptor protein for RNase E and counteraction by an anti-adaptor RNA. *Genes Dev.* **27,** 552–564 (2013).

37. Kalamorz, F., Reichenbach, B., Marz, W., Rak, B. & Gorke, B. Feedback control of glucosamine-6-phosphate synthase GlmS expression depends on the small RNA GlmZ and involves the novel protein YhbJ in Escherichia coli. *Mol Microbiol* **65,** 1518–1533 (2007).

38. Zhang, A. *et al.* Global analysis of small RNA and mRNA targets of Hfq. *Mol Microbiol* **50,** 1111–1124 (2003).

39. Mandin, P. & Gottesman, S. Integrating anaerobic/aerobic sensing and the general stress response through the ArcZ small RNA. *EMBO J.* **29,** 3094–107 (2010).

40. Thomason, M. K., Fontaine, F., De Lay, N. & Storz, G. A small RNA that regulates motility and biofilm formation in response to changes in nutrient availability in Escherichia coli. *Mol Microbiol* **84,** 17–35 (2012).

41. Hemm, M. R. *et al.* Small stress response proteins in Escherichia coli: proteins missed by classical proteomic studies. *J Bacteriol* **192,** 46–58 (2010).

42. Vogel, J., Argaman, L., Wagner, E. G. & Altuvia, S. The small RNA IstR inhibits synthesis of an SOS-induced toxic peptide. *Curr Biol* **14,** 2271–2276 (2004).

43. Darfeuille, F., Unoson, C., Vogel, J. & Wagner, E. G. An antisense RNA inhibits translation by competing with standby ribosomes. *Mol Cell* **26,** 381–392 (2007).

44. Moon, K. & Gottesman, S. A PhoQ/P-regulated small RNA regulates sensitivity of Escherichia coli to antimicrobial peptides. *Mol Microbiol* **74,** 1314–1330 (2009).

45. Udekwu, K. I. *et al.* Hfq-dependent regulation of OmpA synthesis is mediated by an antisense RNA. *Genes Dev* **19,** 2355–2366 (2005).

46. Rasmussen, A. A. *et al.* Regulation of ompA mRNA stability: the role of a small regulatory RNA in growth phase-dependent control. *Mol Microbiol* **58,** 1421–1429 (2005).

47. Coornaert, A. *et al.* MicA sRNA links the PhoP regulon to cell envelope stress. *Mol Microbiol* **76,** 467–479 (2010).

48. Chen, S., Zhang, A., Blyn, L. B. & Storz, G. MicC, a second small-RNA regulator of Omp protein expression in Escherichia coli. *J Bacteriol* **186,** 6689–6697 (2004).

49. Andersen, J. *et al.* The isolation and characterization of RNA coded by the micF gene in Escherichia coli. *Nucleic Acids Res* **15,** 2089–2101 (1987).

50. Andersen, J. & Delihas, N. micF RNA binds to the 5' end of ompF mRNA and to a protein from Escherichia coli. *Biochemistry* **29,** 9249–9256 (1990).

51. Coyer, J., Andersen, J., Forst, S. A., Inouye, M. & Delihas, N. micF RNA in ompB mutants of Escherichia coli: different pathways regulate micF RNA levels in response to osmolarity and temperature change. *J Bacteriol* **172,** 4143–4150 (1990).

52. Holmqvist, E., Unoson, C., Reimegard, J. & Wagner, E. G. A mixed double negative feedback loop between the sRNA MicF and the global regulator Lrp. *Mol Microbiol* **84,** 414–427 (2012).

53. Mandin, P. & Gottesman, S. A genetic approach for finding small RNAs regulators of genes of interest identifies RybC as regulating the DpiA/DpiB two-component system. *Mol Microbiol* **72,** 551–565 (2009).

54. Rasmussen, A. A. *et al.* A conserved small RNA promotes silencing of the outer membrane protein YbfM. *Mol Microbiol* **72,** 566–577 (2009).

55. Figueroa-Bossi, N., Valentini, M., Malleret, L., Fiorini, F. & Bossi, L. Caught at its own game: regulatory small RNA inactivated by an inducible transcript mimicking its target. *Genes Dev* **23,** 2004–2015 (2009).

56. Overgaard, M., Johansen, J., Moller-Jensen, J. & Valentin-Hansen, P. Switching off small RNA regulation with trap-mRNA. *Mol Microbiol* **73,** 790–800 (2009).

57. Kawano, M., Reynolds, A. A., Miranda-Rios, J. & Storz, G. Detection of 5'- and 3'-UTR-derived small RNAs and cis-encoded antisense RNAs in Escherichia coli. *Nucleic Acids Res* **33,** 1040–1050 (2005).

58. Fozo, E. M., Hemm, M. R. & Storz, G. Small toxic proteins and the antisense RNAs that repress them. *Microbiol Mol Biol Rev* **72,** 579–89, Table of Contents (2008).

59. Guillier, M. & Gottesman, S. Remodelling of the Escherichia coli outer membrane by two small regulatory RNAs. *Mol Microbiol* **59,** 231–247 (2006).

60. Guillier, M. & Gottesman, S. The 5' end of two redundant sRNAs is involved in the regulation of multiple targets, including their own regulator. *Nucleic Acids Res* **36,** 6781–6794 (2008).

61. Holmqvist, E. *et al.* Two antisense RNAs target the transcriptional regulator CsgD to inhibit curli synthesis. *EMBO J* **29,** 1840–1850 (2010).

62. De Lay, N. & Gottesman, S. A complex network of small non-coding RNAs regulate motility in Escherichia coli. *Mol. Microbiol.* **86,** 524–38 (2012).

63. Altuvia, S., Weinstein-Fischer, D., Zhang, A., Postow, L. & Storz, G. A small, stable RNA induced by oxidative stress: role as a pleiotropic regulator and antimutator. *Cell* **90,** 43–53 (1997).

64. Altuvia, S., Zhang, A., Argaman, L., Tiwari, A. & Storz, G. The Escherichia coli OxyS regulatory RNA represses fhlA translation by blocking ribosome binding. *EMBO J* **17,** 6069–6075 (1998).

65. Kawano, M., Oshima, T., Kasai, H. & Mori, H. Molecular characterization of long direct repeat (LDR) sequences expressing a stable mRNA encoding for a 35-amino-acid cell-killing peptide and a cis-encoded small antisense RNA in Escherichia coli. *Mol Microbiol* **45,** 333–349 (2002).

66. Kole, R. & Altman, S. Properties of purified ribonuclease P from Escherichia coli. *Biochemistry* **20,** 1902–1906 (1981).

67. Reed, R. E., Baer, M. F., Guerrier-Takada, C., Donis-Keller, H. & Altman, S. Nucleotide sequence of the gene encoding the RNA subunit (M1 RNA) of ribonuclease P from Escherichia coli. *Cell* **30,** 627–636 (1982).

68. Kim, S. & Lee, Y. Function of the repeated sequence in the 3' flanking region of the Escherichia coli rnpB gene on transcription termination and RNA processing. *FEBS Lett* **407,** 353–356 (1997).

69. Majdalani, N., Chen, S., Murrow, J., St John, K. & Gottesman, S. Regulation of RpoS by a novel small RNA: the characterization of RprA. *Mol Microbiol* **39,** 1382–1394 (2001).

70. Majdalani, N., Hernandez, D. & Gottesman, S. Regulation and mode of action of the second small RNA activator of RpoS translation, RprA. *Mol Microbiol* **46,** 813–826 (2002).

71. Mika, F. *et al.* Targeting of csgD by the small regulatory RNA RprA links stationary phase, biofilm formation and cell envelope stress in Escherichia coli. *Mol Microbiol* **84,** 51–65 (2012).

72. Douchin, V., Bohn, C. & Bouloc, P. Down-regulation of porins by a small RNA bypasses the essentiality of the regulated intramembrane proteolysis protease RseP in Escherichia coli. *J Biol Chem* **281,** 12253–12259 (2006).

73. Bosl, M. & Kersten, H. A novel RNA product of the tyrT operon of Escherichia coli. *Nucleic Acids Res* **19,** 5863–5870 (1991).

74. Waters, L. S., Sandoval, M. & Storz, G. The Escherichia coli MntR miniregulon includes genes encoding a small protein and an efflux pump required for manganese homeostasis. *J Bacteriol* **193,** 5887–5897 (2011).

75. Gerstle, K., Klatschke, K., Hahn, U. & Piganeau, N. The small RNA RybA regulates key-genes in the biosynthesis of aromatic amino acids under peroxide stress in E. coli. *RNA Biol* **9,** 458–468 (2012).

76. Johansen, J., Rasmussen, A. A., Overgaard, M. & Valentin-Hansen, P. Conserved small non-coding RNAs that belong to the sigmaE regulon: role in down-regulation of outer membrane proteins. *J Mol Biol* **364,** 1–8 (2006).

77. Thompson, K. M., Rhodius, V. A. & Gottesman, S. SigmaE regulates and is regulated by a small RNA in Escherichia coli. *J Bacteriol* **189,** 4243–4256 (2007).

78. Antal, M., Bordeau, V., Douchin, V. & Felden, B. A small bacterial RNA regulates a putative ABC transporter. *J Biol Chem* **280,** 7901–7908 (2005).

79. Frohlich, K. S., Papenfort, K., Berger, A. A. & Vogel, J. A conserved RpoS-dependent small RNA controls the synthesis of major porin OmpD. *Nucleic Acids Res* **40,** 3623–3640 (2012).

80. Masse, E. & Gottesman, S. A small RNA regulates the expression of genes involved in iron metabolism in Escherichia coli. *Proc Natl Acad Sci U S A* **99,** 4620–4625 (2002).

81. Masse, E., Vanderpool, C. K. & Gottesman, S. Effect of RyhB small RNA on global iron use in Escherichia coli. *J Bacteriol* **187,** 6962–6971 (2005).

82. Prevost, K. *et al.* The small RNA RyhB activates the translation of shiA mRNA encoding a permease of shikimate, a compound involved in siderophore synthesis. *Mol Microbiol* **64,** 1260–1273 (2007).

83. Salvail, H. *et al.* A small RNA promotes siderophore production through transcriptional and metabolic remodeling. *Proc Natl Acad Sci U S A* **107,** 15223–15228 (2010).

84. Vanderpool, C. K. & Gottesman, S. Involvement of a novel transcriptional activator and small RNA in post-transcriptional regulation of the glucose phosphoenolpyruvate phosphotransferase system. *Mol Microbiol* **54,** 1076–1089 (2004).

85. Wadler, C. S. & Vanderpool, C. K. A dual function for a bacterial small RNA: SgrS performs base pairing-dependent regulation and encodes a functional polypeptide. *Proc Natl Acad Sci U S A* **104,** 20454–20459 (2007).

86. Rice, J. B. & Vanderpool, C. K. The small RNA SgrS controls sugar-phosphate accumulation by regulating multiple PTS genes. *Nucleic Acids Res* **39,** 3806–3819 (2011).

87. Pedersen, K. & Gerdes, K. Multiple hok genes on the chromosome of Escherichia coli. *Mol Microbiol* **32,** 1090–1102 (1999).

88. Rice, P. W., Polayes, D. A. & Dahlberg, J. E. Spot 42 RNA of Escherichia coli is not an mRNA. *J Bacteriol* **169,** 3850–3852 (1987).

89. Moller, T., Franch, T., Udesen, C., Gerdes, K. & Valentin-Hansen, P. Spot 42 RNA mediates discoordinate expression of the E. coli galactose operon. *Genes Dev* **16,** 1696–1706 (2002).

90. Beisel, C. L. & Storz, G. The base-pairing RNA spot 42 participates in a multioutput feedforward loop to help enact catabolite repression in Escherichia coli. *Mol Cell* **41,** 286–297 (2011).

91. Vogel, J. *et al.* RNomics in Escherichia coli detects new sRNA species and indicates parallel transcriptional output in bacteria. *Nucleic Acids Res* **31,** 6435–6443 (2003).

92. Hobbs, E. C., Astarita, J. L. & Storz, G. Small RNAs and small proteins involved in resistance to cell envelope stress and acid shock in Escherichia coli: analysis of a bar-coded mutant collection. *J Bacteriol* **192,** 59–67 (2010).

93. Muto, A. *et al.* Structure and function of 10Sa RNA: trans-translation system. *Biochimie* **78,** 985–991 (1996).

94. Wassarman, K. M. & Storz, G. 6S RNA regulates E. coli RNA polymerase activity. *Cell* **101,** 613–623 (2000).

95. Kawano, M., Aravind, L. & Storz, G. An antisense RNA controls synthesis of an SOS-induced toxin evolved from an antitoxin. *Mol Microbiol* **64,** 738–754 (2007).

96. Wassarman, K. M. & Saecker, R. M. Synthesis-mediated release of a small RNA inhibitor of RNA polymerase. *Science.* **314,** 1601–1603 (2006).

97. Raghavan, R., Groisman, E. A. & Ochman, H. Genome-wide detection of novel regulatory RNAs in E. coli. *Genome Res* **21,** 1487–1497 (2011).

98. Chiang, M. K., Lu, M. C., Liu, L. C., Lin, C. T. & Lai, Y. C. Impact of Hfq on global gene expression and virulence in Klebsiella pneumoniae. *PLoS One* **6,** e22248 (2011).

99. Sittka, A. *et al.* Deep sequencing analysis of small noncoding RNA and mRNA targets of the global post-transcriptional regulator, Hfq. *PLoS Genet* **4,** e1000163 (2008).
